# Vineyard Soil Microbiome Composition Related to Rotundone Concentration in Australian Cool Climate ‘Peppery’ Shiraz Grapes

**DOI:** 10.3389/fmicb.2019.01607

**Published:** 2019-07-16

**Authors:** Vadakattu V. S. R. Gupta, Robert G. V. Bramley, Paul Greenfield, Julian Yu, Markus J. Herderich

**Affiliations:** ^1^CSIRO Agriculture and Food, Urrbrae, SA, Australia; ^2^CSIRO Energy, North Ryde, NSW, Australia; ^3^School of Life Sciences, Arizona State University, Mesa, AZ, United States; ^4^The Australian Wine Research Institute, Urrbrae, SA, Australia

**Keywords:** rotundone, microbiome diversity, bacteria, fungi, grapes

## Abstract

Soil microbial communities have an integral association with plants and play an important role in shaping plant nutrition, health, crop productivity and product quality. The influence of bacteria and fungi on wine fermentation is well known. However, little is known about the role of soil microbes, other than microbial pathogens, on grape composition or their role in vintage or site (*terroir*) impacts on grape composition. In this study, we used an amplicon sequencing approach to investigate the potential relationships between soil microbes and inherent spatial variation in grape metabolite composition – specifically, the concentration of the ‘impact aroma compound’ rotundone in Shiraz grapes (*Vitis vinifera* L.) grown in a 6.1 ha vineyard in the Grampians region of Victoria, Australia. Previous work had demonstrated temporal stability in patterns of within-vineyard spatial variation in rotundone concentration, enabling identification of defined ‘zones’ of inherently ‘low’ or ‘high’ concentration of this grape metabolite. 16S rRNA and ITS region-amplicon sequencing analysis of microbial communities in the surface soils collected from these zones indicated marked differences between zones in the genetic diversity and composition of the soil bacterial and fungal microbiome. Soils in the High rotundone zone exhibited higher diversity of bacteria, but lower diversity of fungi, compared to the soils in the Low rotundone zone. In addition, the network analysis of the microbial community in the High rotundone zone soils appeared well structured, especially with respect to the bacterial community, compared to that in the Low rotundone zone soils. The key differences in the microbial community structure between the rotundone zones are obvious for taxa/groups of both bacteria and fungi, particularly for bacteria belonging to Acidobacteria-GP4 and GP7, Rhizobiales, Gaiellaceae, Alphaproteobacteria and the Nectriaceae and Tremellaceae families of fungi. Although mulching in some parts of the vineyard caused changes in bacterial and fungal composition and overall microbial catabolic diversity and activity, its effects did not mask the rotundone zone-based variation. This finding of a systematic rotundone zone-based variation in soil microbiomes suggests an opportunity to bring together understanding of microbial ecology, plant biochemistry, and viticultural management for improved management of grape metabolism, composition and wine flavor.

## Introduction

**FIGURE 1:**
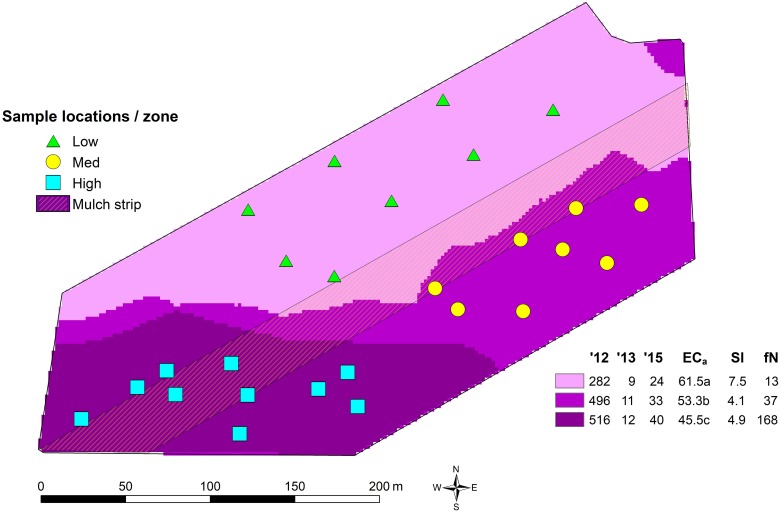
Soil sampling locations, rotundone zones and the mulched strip in the 6.1 ha study vineyard. The rotundone zones of Bramley et al. (2017) were identified through *k*-means clustering of map layers of grape berry rotundone concentration in 2012 (’12), 2013 (’13), and 2015 (’15) along with apparent electrical soil conductivity (EC_*a*_), slope (Sl) and aspect, expressed in terms of orientation away from north (fN). The values in the legend are the zone means. Note that the overall block mean berry rotundone concentration varied by a factor of 40-fold over the 3 years in which rotundone concentrations were mapped. Bramley et al. (2017) provide further details.

Plant–microbe interactions are both dynamic and complex in terms of beneficial and deleterious associations which play a key part in plant growth, tolerance against stresses, nutrition, productivity and product quality (Gupta et al., 2011; Pieterse et al., 2016). The soil on which vines are grown has been suggested to impart a unique quality to the grapes and wine due to the physiological responses of the vines to soil type, topography and climatic conditions, in addition to their viticultural management (Gilbert et al., 2014; Zarraonaindia et al., 2015; Castaneda and Barbosa, 2017). Thus, a wines’ *terroir* (e.g., van Leeuwen and Seguin, 2006) or *sense of place* (Goode, 2005) is a reflection of both the biophysical and social conditions in which the grapes were grown and wine made. In this context, the ‘spicy,’ ‘peppery’ flavor and aroma of some cooler climate Australian Shiraz wines has been suggested as evocative of their *terroir* (Herderich et al., 2012), a characteristic which has also been noted in other cooler climate wines made from other grape varieties (e.g., Geffroy et al., 2014). This ‘pepperiness’ is due to the presence of rotundone, a grape-derived sesquiterpene (Siebert et al., 2008; Wood et al., 2008) and has been found to be consistently pronounced in Shiraz wine produced in the cool Grampians region of Victoria, Australia (Jeffery et al., 2009). Recent research conducted at the within-vineyard scale (Scarlett et al., 2014; Bramley et al., 2017) has demonstrated that variation in the concentration of rotundone in grape berries is spatially structured and related to variation in the land underlying the vineyard, with the patterns of spatial variation being stable between seasons, in spite of marked annual variation in the mean rotundone concentration. Thus, it was possible to identify ‘zones’ within the vineyard in which the concentration of rotundone in grape berries was characteristically ‘lower’ or ‘higher’ (Bramley et al., 2017; Figure 1). Although variation in soil and topography (in particular, aspect, which affects temperature and/or solar radiation) have been proposed as strong drivers for within-vineyard variation in the rotundone concentration (Scarlett et al., 2014; Bramley et al., 2017), the contribution of specific soil physical, chemical and/or biological factors is not known.

In general, the formation of sesquiterpenes can be a typical plant metabolic response to herbivore action or other stress factors (Schuman and Baldwin, 2018). However, the consistent spatial pattern of High and Low rotundone zones across a large site and multiple vintages pointed toward a stationary effector in the vineyard studied. One such trigger was speculated to be variation in the vineyard soil microbiome which can play specific roles in the productivity and disease incidence and resistance of a host plant (Deyett et al., 2017). It has also been suggested to influence, directly or indirectly, fruit development and health which, in turn, may impact on fruit quality (Barata et al., 2011; Gilbert et al., 2014; Mezzasalma et al., 2017; Stefanini and Cavalieri, 2018). Recent evidence has emerged that the majority of bacterial taxa associated with grape berries originates from the soil, and that their distribution reflects the influence of highly localized biogeographic factors and vineyard management (Zarraonaindia et al., 2015).

Plant roots are colonized by a subset of organisms from the soil microbiome creating rhizosphere and endosphere communities enriched with specific species (Pieterse et al., 2016). Typically, soil microbiome composition depends on both the abiotic (e.g., pH, organic C and C:N ratio) and biotic (plant type) factors, and variations in these can cause differences in biological functioning and, in turn, crop productivity (Fierer and Jackson, 2006; Penton et al., 2014; Zarraonaindia et al., 2015; Fierer, 2017). However, the root and endophytic communities from different soils contain overlapping and low complexity communities which are enriched by specific members of soil bacteria (Shade and Handelsman, 2012; Gupta et al., 2014; Bai et al., 2015; Bulgarelli et al., 2015; Zarraonaindia et al., 2015; Fierer, 2017).

Distinct bacterial, fungal and yeast communities were found to be associated with vineyard soil, root, leaves, grapes, flowers, and grape juice (Zarraonaindia et al., 2015; Wei et al., 2018), yet their impact (if any) on grape metabolism is not understood. The natural berry microbiome is influenced by pedoclimatic factors and management; some members of a berry’s microbiome that persist in grape juice can influence the fermentation process and wine quality (Martins et al., 2012; Mezzasalma et al., 2017). Grape health status has been suggested to be the main factor affecting the microbial ecology of grapes (Barata et al., 2011). It has also been shown that the bacterial and fungal taxonomic structure of wine-grape surfaces or spontaneous wine fermentations can be distinctive, supporting the notion that regional based variation in microbial communities can have an impact on a wine’s sensory attributes (Bokulich et al., 2014; Sternes et al., 2017). Overall, these observations suggest that differences in the vineyard soil microbiome in terms of its structure, diversity and microbial activity could potentially contribute to variation in grape and wine composition, sensory characteristics and a wines’ *terroir*, through either the vine’s response to its specific soil microbiome and/or through the soil microbiome shaping the grape microbiome and subsequent fermentation outcomes.

In the present study, we characterized the soil microbiome communities (bacteria and fungi) in the different rotundone concentration-based zones within the Grampians vineyard studied by Scarlett et al. (2014) and Bramley et al. (2017). Specifically, our aims were to see whether the soil microbiome composition and diversity differed between the High and Low rotundone zones of Bramley et al. (2017; Figure 1) and to characterize bacterial and fungal communities in an Australian vineyard soil. We also sought to assess the potential effects of management on the soil microbiome through the application of mulch. For this, surface (0–15 cm) soils were analyzed using 16S rRNA (bacteria) and ITS-region (fungi) amplicon sequencing and qPCR methods to describe the composition and abundances of bacteria and fungi along with physico-chemical analysis of soil properties. Microbial activity and catabolic diversity in terms of multiple C substrate utilization profiles using a modified Microresp^®^ technique were also assessed to determine the effects of mulch application.

## Materials and Methods

### Vineyard Details

The 6.1 ha vineyard is located at the Mount Langi Ghiran vineyard in the Grampians region of Victoria, Australia (37°S, 143°E). It was planted to Shiraz grapes on own roots in 1968. The soils in the block may be characterized as duplex, predominantly silty loams over clays with some areas of sandier soils. Details of the vineyard climate, soils, management and production focus are given in Scarlett et al. (2014) and Bramley et al. (2017) who also describe the delineation of the ‘rotundone zones’ (Figure 1). Of particular relevance here was the application of mulch in August 2016 to part of the vineyard (Figure 1) as a part of normal commercial operations. Oaten straw mulch was applied on the surface to a ten row strip (approximately 1.3 ha) at a rate of approximately 28 t/ha with the aim of buffering against hot weather and improving water use efficiency in the face of reduced water supply due to drought.

### Soil Sample Collection and Preparation

On March 27th and 28th, 2017 (i.e., 7–8 months after mulch application), surface soils (0–5 and 5–15 cm) were collected in each of the previously described rotundone zones *viz*. Low, Medium, and High (Bramley et al., 2017; Figure 1). The samples were collected from positions adjacent to selected geo-referenced vines from the Scarlett et al. (2014) and Bramley et al. (2017) studies. Prior to the collection of soils, any surface plant residues were removed and, using a 2.5 cm diameter soil corer, were separated into the two depths (Supplementary Figure S1). Five soil cores were collected adjacent to each vine (within 50 cm of the trunk) and mixed to give a combined sample. A minimum of five samples were collected in each of the rotundone zones. Additionally, in order to determine the effect of surface mulching in the Medium and High rotundone zones, we also collected samples from the mulched areas after removing the surface mulch (Supplementary Figure S1). All samples were stored in a cool box with icepacks for transport to the laboratory. Prior to laboratory analysis all soils were sieved through a 2 mm sieve to remove stones and undecomposed organic material and subsamples for DNA extraction were stored at −20°C.

### Chemical and Microbial Activity Measurements

The collected soil samples were sub-divided for chemical and microbial activity analysis. Those used for chemical analysis were air dried at 40°C prior to analysis, whereas field moist samples were used in the microbial activity measurements. Analyses for various chemical properties were conducted using established standard methods described in Rayment and Lyons (2011) in an appropriately accredited commercial soil testing laboratory^1^. Soil texture was determined using a combination of micropipette (Miller and Miller, 1987) and MIR methods (Janik et al., 2009). Total nitrogen levels were measured using a LECO TruMac CN Macro Determinator^2^. All results are expressed on per gram dry soil basis and all samples were analyzed in-duplicate. All results are presented in Table 1 and Supplementary Table S1.

**TABLE 1:** Physical and chemical properties of soils.

**Property**	**Low**	**Medium**	**High**	***F*-test**	**LSD (*P* < 0.05)**
pH (water)	6.9	6.7	7.0	NS	
Organic carbon (%)	2.0	2.3	1.8	NS	
TN (μg/g)	8.6	5.0	5.3	NS	
DOC (μg/g)	26.0	47.9	19.5	0.00	15.1
MinN (μg/g)	6.3	2.0	3.7	0.003	3.2
Colwell P (μg/g)	11.3	15.5	26.9	0.001	7.1
Colwell K (μg/g)	171.0	249.6	130.0	0.001	1.3
KCL sulfur (μg/g)	7.5	8.9	5.6	NS	
DTPA-Cu (mg/kg)	6.9	7.2	12.0	NS	
DTPA-Zn (mg/kg)	2.1	3.7	5.9	NS	
DTPA-Mn (mg/kg)	1.8	2.7	1.7	0.046	0.7
DTPA-Fe (mg/kg)	30.6	37.4	26.1	NS	
Exchangeable CEC (c.mol/kg)	9.0	9.6	7.7	NS	
Clay (%)	5.3	6.2	3.6	0.006	1.7
Sand (%)	42.6	39.7	61.1	0.001	6.7
Silt (%)	52.1	54.1	35.4	0.001	5.8

Microbial catabolic response (CO_2_ production to the addition of specific C-containing substrates) and diversity was measured through carbon substrate utilization profiles of soil microbial communities (‘community-level physiological profiles,’ CLPP) using a modified Microresp^®^ technique (Campbell et al., 2003) adjusted for Australian soils (Knox et al., 2009). The average metabolic response (AMR) reflecting the overall functional capability of soil heterotrophic microbial communities and community metabolic diversity (CMD) was estimated based on substrate induced respiration and number of substrates utilized.

Rotundone in grapes sampled from within the Low and High zones prior to harvest in 2017 was quantified as described before (Bramley et al., 2017); the data confirmed the concentration differences between Low and High rotundone zones observed in earlier vintages at the date of the soil sampling (March 28, 2017, average 47 ng/kg for 6 grape samples from the Low zone and 90 ng/kg for grapes from the High zone). The spatially structured rotundone concentration differences continued to increase until full maturity (April 18, 2017, average 173 ng/kg for 6 grape samples from the Low zone and 321 ng/kg for grapes from the High zone).

### DNA Extraction

DNA was extracted from 2.5 g samples of soil using the DNeasy PowerMax soil kit^3^ following the manufacturer’s protocol. Mechanical disruption of the soil using bead-beating (speed 4.5, 30 s; FP120; Qbiogene Inc., Carlsbad, CA, United States) was applied and the final DNA extracts were eluted (2×) using 1 ml of warmed (60°C) C6 solution two times (5 min each) to maximize DNA yield (a final volume of 2 ml) and the extracts stored at −80°C. DNA extracts were also further cleaned using MinElute 96 UF PCR Purification Kit^4^ and DNA eluted into nuclease free water.

### Bacteria (16S rRNA) and Fungi (ITS Region) Amplicon Sequencing

For bacterial and fungal community composition analysis, 16S rRNA and ITS region PCR amplification and sequencing was conducted using the primers for V1–V3 16S region (27F-519R) and for ITS1F-2R, respectively. Briefly, PCR amplicons were generated with the group specific primers and conditions outlined in Table 1, using AmpliTaq Gold 360 mastermix (Life Technologies, Australia) for the primary PCR. A secondary PCR to index the amplicons was performed with TaKaRa Taq DNA Polymerase (Clontech, Vic, Australia). The resulting amplicons were measured by fluorometry (Invitrogen Picogreen, MA, United States) and normalized. Application of 12 bp barcodes, clean up of PCR products and the preparation of libraries for sequencing were done following standard Illumina Miseq protocols^5^. The eqimolar pool was then measured by qPCR (KAPA) followed by sequencing on the Illumina MiSeq (San Diego, CA, United States) with 2 × 300 base pairs paired-end chemistry^6^.

### Amplicon Sequence Data Analysis

The initial amplicon sequence data was processed using GHAP (Greenfield, 2017), an in-house amplicon clustering and classification pipeline built around tools from USearch (Edgar, 2013) and the RDP^7^ (Cole et al., 2014), combined with locally written tools for demultiplexing and generating OTU tables. This hybrid pipeline takes files of reads and produces tables of classified OTUs (Operational Taxonomic Units) and their associated reads counts across all samples. The amplicon reads are demultiplexed, and the read pairs are then merged and de-replicated.

For bacterial 16S rRNA gene amplicon reads, the merged reads are then trimmed and clustered at 97% similarity to generate OTUs. Representative sequences from each OTU are then classified both by finding their closest match in a set of reference 16S sequences, and by using the RDP Naïve Bayesian Classifier. The pipeline provides both the RDP 16S Training Set and the RefSeq 16S reference sequence collection for the purposes of species-level classification, although any reference collection can be used and the provided sets can easily be customized by adding further genes.

Fungal ITS regions are quite variable in length and can be longer than can be completely covered by a pair of reads. A conventional merge step will fail for such organisms as it depends on each pair of reads having a sufficiently long (and similar) overlapping region. Such merging problems can result in the disappearance of whole classes of organisms from the final OTU tables. The pipeline handles this situation by using the forward (R1) reads in those cases where the merging step failed to find a good overlapping region for a read pair.

The combined merged reads are then trimmed and clustered at 97% similarity to generate OTUs. Representative sequences from each OTU were then classified both by finding their closest match in a set of reference fungal ITS sequences, and by using the RDP Naïve Bayesian Classifier with the Warcup training set (Deshpande et al., 2016). The pipeline uses the Warcup fungal ITS training set for the purposes of species-level classification, although any reference collection could be used and the Warcup reference set can be easily be customized by adding further genes.

The pipeline then maps the merged reads back onto the classified OTU sequences to get accurate read counts for each OTU/sample pairing, and generates OTU tables in both text and .biom (v1) formats, complete with taxonomic classifications and species assignments. The OTU tables are then summarized over all taxonomic levels, combining the counts for identified taxa across all OTUs. The pipeline finally classifies all the merged reads using the RDP Classifier, regardless of whether they were assigned to an OTU. This last step is done to provide confidence in the clustering and OTU formation steps by providing an independent view of the community structure.

Venn diagrams were generated to assess the distinct and common bacterial and fungal OTUs among different rotundone zones; for this, OTUs had to occur in at least 5 samples.

### Gene Abundance Using qPCR

DNA in each sample was quantified against a DNA standard (λ-phage DNA; *R*^2^ = 0.98) using the Quant-iT PicoGreen dsDNA assay (Invitrogen, MA, United States). The final extracted DNA was diluted 1:10 to a final volume of 50 μL in molecular grade H_2_O and 3 μL was used per 15 μL PCR reaction. Amounts of total fungal and bacterial abundances were quantified using group specific primers [Fungi – FR1/FF390 (TTGGTCATTTAGAGGAAGTAA/TTYGCTGYGTTCTTCAT CG; Vainio and Hantula, 2000); Bacteria-F968/R1378 (AACG CGAAGAACCTTAC/CGGTGTGTACAAGGCCCGGGAACG; Smalla et al., 2007] based on the chemistry from the QuantiTect SYBR Green PCR kit (Qiagen, VIC, Australia) and the PCR was carried out on a Strategene Maxpro3000P qPCR system (Agilent, VIC, Australia). qPCR was performed against a standard curve of known 16S and ITS gene copies. For bacterial qPCR, bacterial DNA amplified from soil DNA by using the same primers was cloned into the pGEM-T easy vector, and a standard curve containing a known amount of bacteria DNA was used to calculate the number of 16S rRNA gene copies in the soil samples. For fungal qPCR, known amounts of culture DNA from *Rhizoctonia*, *Fusarium*, and a *Trichoderma* isolate were combined to make a standard curve for quantification of fungal DNA in the samples.

Briefly, for bacterial qPCR the PCR conditions were 95°C for 15 min initial denaturation followed by 40 cycles of 95°C for 30 s, 54°C for 30 s, and 72°C for 1 min. The melt curve for the amplified products was carried out at 54–95°C at 0.5°C increments for 5 s. For fungal qPCR, PCR conditions were 95°C for 15 min initial denaturation followed by 45 cycles of 95°C for 10 s, 50°C for 10 s, and 72°C for 40 s. Melt curve for the amplified products was carried out by 50–95°C at 0.5°C increments for 5 s.

### Molecular (RMT) Network Analysis

To decipher microbial community co-occurrence patterns, molecular ecological networks, based on statistical correlations, for bacterial and fungal communities were constructed based on 16S rRNA gene and ITS-region amplicons. Individual networks were constructed for rotundone Low, rotundone High and rotundone High without mulch. Henceforth, the bacterial networks were referred to as 16S-low, 16S-high and 16S-highNM networks, and fungal networks referred to as ITS-low, ITS-high, and ITS-highNM networks. The OTU tables were separated by rotundone zone and trimmed to remove singleton/doubletons. The resulting OTU tables used for bacterial networks contained 5628 OTUs for 16S-high and 16S-highNM networks and 4,318 OTUs for the 16S-low network. Fungal networks were based on 1,101 OTUs, 799 OTUs, 1,304 OTUs in the ITS-high, ITS-highNM, and ITS-low network, respectively. Networks were constructed using the Molecular Ecological Network Analysis (MENA) Pipeline^8^ (Deng et al., 2012). MENA was implemented with random matrix theory (RMT) based methods to automatically identify the appropriate similarity threshold, the minimum strength of the connection between each pair of nodes, prior to network construction (Zhou et al., 2010, 2011; Deng et al., 2012). Global network properties were calculated, including indexes of individual nodes, modules and interactions, which were subsequently visualized using Cytoscape (Shannon et al., 2003). In network analysis, a group of nodes (i.e., OTUs) highly connected among nodes of the group, but much less connected to nodes outside the group, is defined as a module (Newman, 2006). Here, modules were detected using the leading eigenvector method for module separation and modularity calculations with a modularity threshold of 0.4 to define modular structures in the network (Newman, 2006). Network modules were grouped by modularity (i.e., module number) with the size of the node corresponding to connectivity (i.e., node degree). Node connectivity within a module (Zi) and between modules (Pi) coefficients were used to determine the node topology. Node topologies were organized into four categories: network hubs (Zi > 2.5, Pi > 0.62), module hubs (Zi > 2.5, Pi ≤ 0.62), connectors (Zi ≤ 2.5, Pi > 0.62) or peripherals (Zi ≤ 2.5, Pi ≤ 0.62) (Olesen et al., 2007; Zhou et al., 2010; Deng et al., 2012). Determination of higher order organization of the modules in the network and the correlations of the modules to soil chemical properties was carried out using Module-Eigengene analysis, modules with less than five nodes were excluded.

### Statistical Analysis

We used several multi-variate statistical analyses for community comparisons. To avoid effects of sequencing depth based biases, the bacterial and fungal sequence data were, respectively, rarified to even depths of 27,500 and 35,000 sequences per sample, prior to community analysis. Raw cluster abundances were Hellinger transformed and a Bray–Curtis dissimilarity matrix (+1) was constructed, statistical analyses performed and diversity estimates calculated using PRIMER-E (Primer 7, Clarke and Gorley, 2006). Cluster analysis was performed with the Similarity Profile analysis (SIMPROF) test (Clarke et al., 2008). Significant differences in community structure were tested for zones, zone × depth and mulch × depth models with Permutational Multivariate Analysis of Variance (PERMANOVA) (Anderson, 2001) and Analysis of Similarity (ANOSIM) (Clarke and Ainsworth, 1993). Canonical analysis on principle co-ordinates (CAP) and distance based Redundancy analysis (dbRDA) were performed for factor groups that were found significant with PERMANOVA only. BIO-ENV test (biota and/or environment matching; Clarke and Ainsworth, 1993) was done to find any links between multivariate community structure and soil physico-chemical properties. ANOVA statistics for Shannon diversity (*H*), Pielou’s Evenness (*J*), Margalef’s Richness (*d*), and the number of individuals (*N*) were performed using Minitab 16 (Minitab Inc., State College, PA, United States). Additionally, specific members of bacterial and fungal community that were contributing to the dissimilarity between rotundone zones, soil depths and mulching effects were identified through analysis using STAMP software based on Welch’s *t*-test (Parks et al., 2014).

Differences in the relative abundances of bacteria and fungi and diversity measures for different zones, soil depths and mulching treatments were compared by ANOVA analysis using Genstat (v18.1.0, VSN International Ltd.).

## Results

### Soil Physico-Chemical Properties

In general, there were few significant differences between the different rotundone zones in soil physico-chemical properties such as soil pH, organic C, total N (Table 1 and Supplementary Table S1). Colwell P was significantly higher and nitrate N, KCl extractable S and EC were lower in the High rotundone zone soils compared to that in the Low rotundone zones. Surface (0–5 cm) soils generally exhibited significantly higher organic C, total N, Ca:Mg ratio and higher pH values compared to the 5–15 cm soils (Table 1 and Supplementary Table S1). Mulching showed no significant effect on total soil organic C, N, and pH levels, but increased exchangeable K, DTPA-extractable Mn and dissolved organic C. There were some minor, but significant, differences in the % sand and % silt levels in soils from High and Low rotundone zones; soils from High rotundone zone had higher silt content (61%) compared to Low and Medium zone soils (43 and 39%, respectively).

### Sequence Analysis

A total of 2,181,623 16S rRNA and 4,327,722 ITS region quality filtered amplicon sequences were obtained from the 52 soil samples with averages of 41,954 and 83,225 sequences per sample for bacteria and fungi, respectively (Supplementary Table S2). Rarefaction curves showed saturation in terms of number of OTUs vs. sequences for both bacteria and fungi (data not shown). Thus, the sequencing depth was considered adequate to cover the full soil microbial community. Following clustering and removal of mitochondria and chloroplast related sequences there were 3,443 and 482 bacterial and fungal OTUs per sample, respectively.

The number of OTUs were significantly (*P* < 0.014) higher for the surface 0–5 cm soil compared to that for the 5–15 cm soil but no significant differences in OTU numbers were observed between no-mulch and mulch samples (Supplementary Table S2). Mulching increased fungal OTUs in the High rotundone zone (443 ± 52 vs. 326 ± 22 OTUs in the Mulch and no-mulch samples). Mulching reduced the number of bacterial OTUs but the effect was mainly seen in the surface 0–5 cm soil in the High rotundone zone only (3,961 ± 69 vs. 3,191 ± 252 in the no-mulch and mulch soils, respectively).

### Diversity and Abundance of Bacteria and Fungi

**FIGURE 2:**
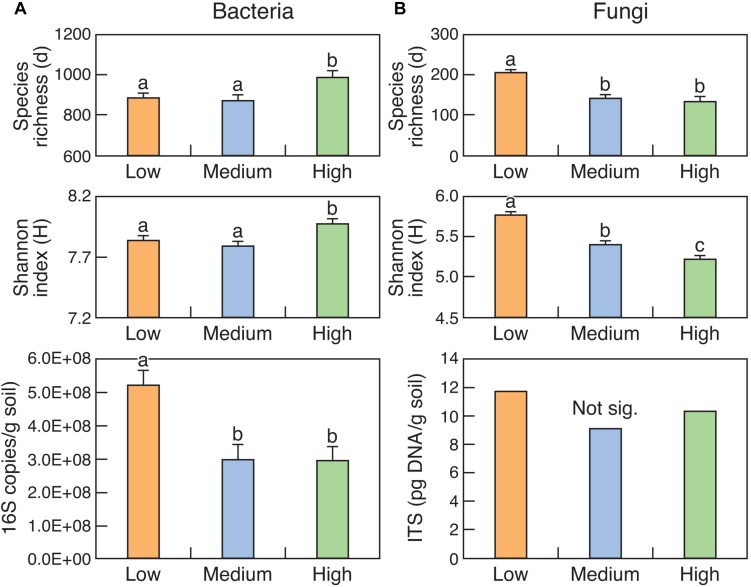
Diversity and abundance of bacteria (16S rRNA) **(A)** and fungi (ITS region) **(B)** in surface (0–15 cm) soils from the three rotundone-based zones. Bars with different alphabets are significantly different from each other at *P* < 0.05.

The abundance of bacteria (16S rRNA copy number) was significantly (*P* < 0.05) higher in the Low rotundone zone compared to that in the Medium and High rotundone zones (Figure 2). The trends were the same for fungi but were not significant. Diversity indices like Margalef’s species richness and Shannon index indicated that the diversity of bacteria was higher in the High rotundone zone compared to that in the Low and Medium zones but the trend was opposite for the fungal community (Figure 2). In general, the abundance and diversity of bacteria was higher in the surface 0–5 cm soil compared to that in the 5–15 cm soil (Supplementary Table S4). Although the diversity of fungi was higher in the surface 0–5 cm, the differences in the abundance were not significant. Mulching generally decreased bacterial diversity whereas it increased the diversity of fungi, with this effect seen mainly in the surface 0–5 cm soil in the High rotundone zone (Supplementary Table S4).

**FIGURE 3:**
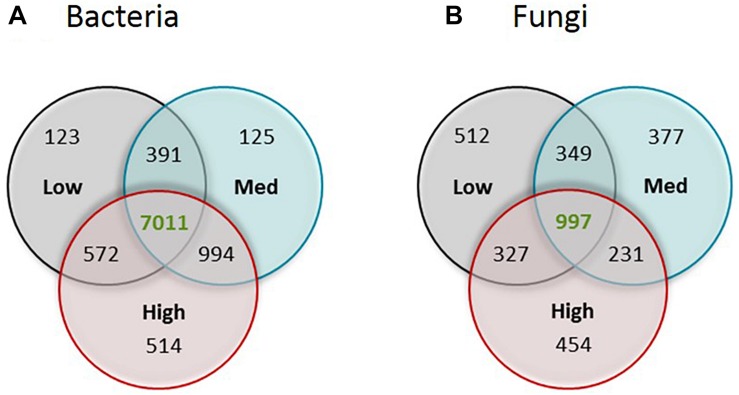
Venn diagram showing the number of unique OTUs of **(A)** bacteria and **(B)** fungi for each of the rotundone zone and shared between the three rotundone-based soil zones.

Venn diagrams were generated to assess the distinct and common bacterial and fungal OTUs among different rotundone zones (Figure 3); for this OTUs had to occur in at least 5 samples. There were 514 bacterial and 454 fungal OTUs that were specific to the High rotundone zone soils only. Similarly, 123 bacterial and 512 fungal OTUs were present in the Low rotundone zone only. 572 bacterial OTUs and 327 fungal OTUs were common between the Low and High rotundone zones, whereas the core microbiome for this vineyard soil, defined by the group of microbes commonly found in all the samples, contained 7,011 bacterial OTUs and 997 fungal OTUs (Figure 3).

### Bacterial and Fungal Community Structure in Different Rotundone Zones

#### Bacteria

**FIGURE 4:**
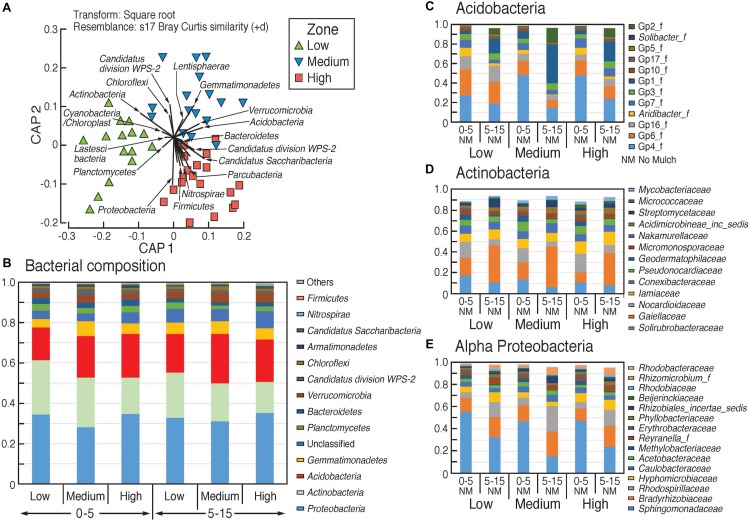
Composition of bacterial communities in the surface soils in the different rotundone-based soil zones. **(A)** Canonical analysis of principle (CAP) ordination, constrained by zone; relative abundances **(B)** at phylum level, **(C–E)** at family level within selected phyla showing significant variation between zones.

Comparison of bacterial community composition from beta-diversity analysis (generated using the Bray–Curtis distance metric) showed significant dissimilarity between the three rotundone zone samples (Figure 4A). Bacteria belonging to the phylum Proteobacteria were the most dominant group (33.3 ± 0.9%) among the 13 phyla of total bacterial community and α-proteobacteria showed significant variation between the different rotundone zones (Figure 4). At the Genus level, a total of 512 bacterial genera (corresponding to 65 ± 0.1% of total sequences) were detected in all the soil samples. Other well-represented phyla included Actinobacteria (20.7 ± 1.1), Acidobacteria (20 ± 0.8%), Gemmatimonadetes (5.8 ± 0.3%), Verrucomicrobia (3.5 ± 0.2%), and Planctomycetes (3.2 ± 0.3%) (Figure 4B). At the family level, bacteria belonging to the 20 most abundant families accounted for 74% of the total bacterial community which included Gemmatimonadaceae, GP4, Sphingomonadaceae, GP6, Gaiellaceae, Bradyrhizobiaceae etc. (Figure 4C).

At the genus level, Low rotundone zone soils could be discriminated from High rotundone zones based on differences (Two-sided Welch’s test, *P* < 0.05) in the relative abundances of bacterial genera GP4, GP7, *Rhizomicrobium*, *Rhizomicrobium*, GP16, *Solirubrobacter*, *Gaiella*, *Conexibacter*, *Sphingomonas*, GP6, GP16, *Nocardiodes* and some unclassified members of Alphaproteobacteria, Chitinophagaceae, and Rhodospirillaceae (Figure 6). PERMANOVA analysis showed that rotundone zone based variance explained 16.6 and 17.2% of variation (*P* = 0.001) among samples when constraining the analysis by soils, either for both depths together or for individual depths (Supplementary Table S3). Soil depth explained 22.6% of variance in bacterial composition when all the samples were considered (*P* = 0.001). In spite of the significant variation in bacterial composition by depth, depth did not mask the rotundone zone based dissimilarity (ANOSIM Global *R* = 0.844; *P* = 0.01). Results from the SIMPER analysis showed that at the phyla level, dissimilarity in Actinobacteria (16%), Acidobacteria (8.9%), and Gemmatimonadetes (5.5%) mostly contributed to the variation between the Low and High rotundone zones. Additionally, High rotundone soils from no-mulch areas showed significantly higher relative abundances of GP4, GP7, Rhizobiales, and unclassified Alphaproteobacteria, whereas the Low rotundone zones had higher relative abundances of Gaiellaceae, GP6, GP15, Solirubrobacteraceae, Plantomycetaceae and Sphingomonadaceae families (Figure 4). Bacteria belonging to Orders Sphingomonadales, Actinomycetales, Acidobacteria GP1, GP2, GP4, Solirubrobacterales, Gaiellales accounted for most of the significant variation between the two soil depths (Two-sided Welch’s test, *P* < 0.05).

**FIGURE 5:**
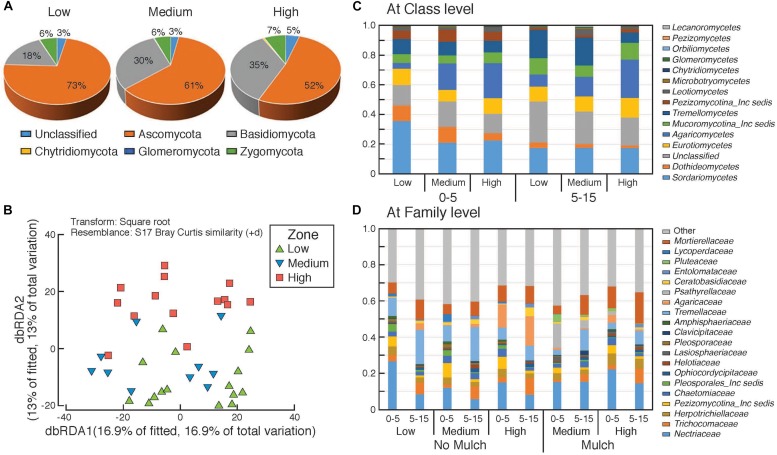
Composition of fungal communities in the surface soils in the different rotundone-based soil zones. Panel **(A)** at Phylum level, **(B)** distance based redundancy analysis (dbRDA); relative abundances **(C)** at Class level and **(D)** at family level showing variation between zones and depths.

**FIGURE 6:**
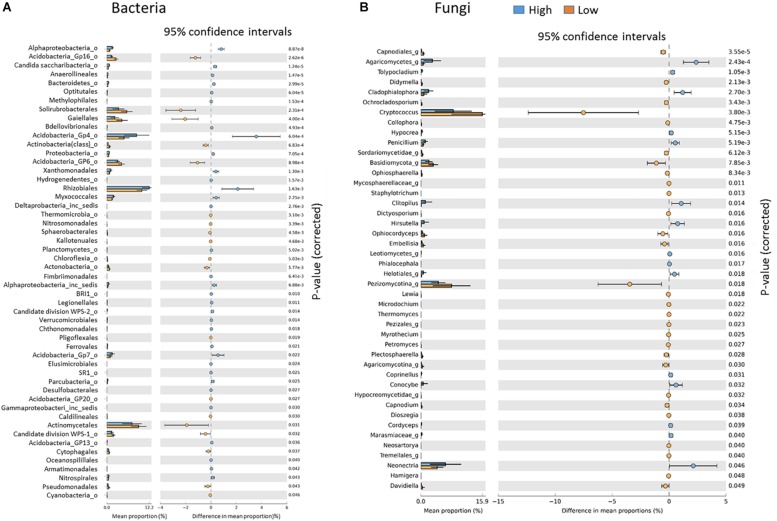
Key indicator bacterial **(A)** and fungal **(B)** groups significantly different between the Low and High rotundone zones.

**FIGURE 7:**
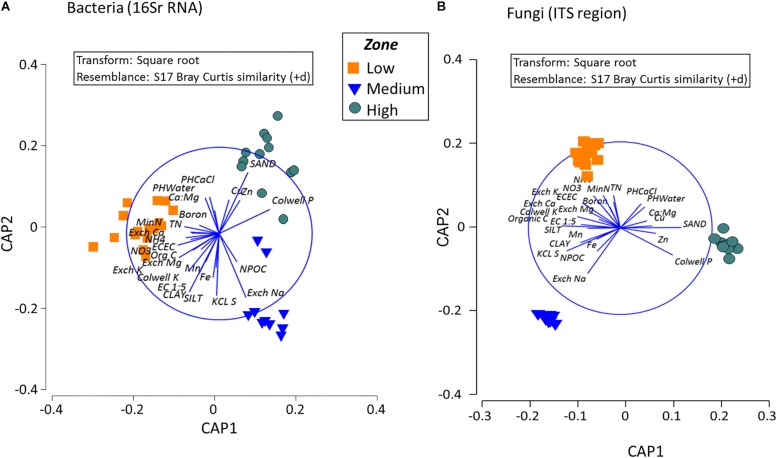
Composition of bacterial and fungal communities in the surface soils in the different rotundone-based soil zones. Canonical analysis of principle (CAP) ordination for **(A)** Bacteria and **(B)** Fungi, constrained by zone based on the Bray–Curtis similarity distance metrics. Vectors in the circle represent fitted values of soil properties showing Pearson correlations *r* > 0.50.

Analysis to identify relationships between the variation in bacterial community in the rotundone zones and soil physico-chemical properties showed significant links between community composition and soil pH, exchangeable Ca, DTPA-extractable Mn, Exch. Na, % sand (ρ = 729, *P* = 0.01). Additionally, application of DistLM analysis (distance based linear models) indicated that soil variables including pH, exchangeable Zn, plant-available Colwell P, exchangeable Na and Mg contributed significantly (*R*^2^ = 0.514; *P* = 0.001) to the zone based variation (Figure 7).

#### Fungi

There was a significant dissimilarity in terms of fungal community composition, from beta-diversity analysis generated using the Bray–Curtis distance metric, between the three rotundone zones (Figure 5B). Fungal species belonging to 5 phyla with 303 genera were identified in all soil samples. Ascomycetes and Basidiomycetes were the predominant fungal phyla in both the surface 0–5 and 5–15 cm soils accounting for 84% of the identified species and on average only 6 ± 1.1% of the OTUs were unclassified (Figure 5A). Fungal species belonging to the phylum Glomeromycota, the mycorrhizal fungi, were generally <0.5% in soils from both depths. Sordariomycetes, Agaricomycetes, Tremellomycetes, Eurotiomycetes were the predominant classes with families Nectriaceae, Tremellaceae, Mortierellaceae, Trichocomaceae, and Agaricaceae showing significant variation between zones (Figures 5C,D).

PERMANOVA analysis showed that rotundone zone based variance explained 16.6 and 17.2% of variation among samples (*P* = 0.001) when constraining the analysis by soils for both depths and individual depths, respectively (Supplementary Table S3). Similar to bacterial community composition, in spite of the significant variation in fungal composition by depth, the rotundone zone based dissimilarities were not masked (ANOSIM Global *R* = 0.683; *P* = 0.01). Soil depth explained 20% of the variance in fungal composition when all the samples were considered (*P* = 0.001; ANOSIM Global *R* = 0.565; *P* = 0.001). In the no-mulch soils, fungi belonging to the Classes Agaricomycetes and Eurotiomycetes were significantly higher in the High rotundone zone soils, whereas Sordariomycetes, Tremellomycetes, and Dothideomycetes were higher in the Low rotundone zone soils (Two-sided Welch’s test, *P* < 0.05).

Fungal taxa associated with the family Tremellaceae and Agaricomycetes were significantly different between the High and Low rotundone soils (Two-sided Welch’s test, *P* < 0.05). Differences in relative abundances of fungal genera *Neonectria*, *Cladophialophora*, *Clitopilus*, *Penicillium*, *Cryptococcus* and Pezizomycotinia, Agaricomycetes, and Basidiomycota contributed to the dissimilarity between Low and High rotundone zone soils (Two-sided Welch’s test, *P* < 0.05; Figure 6B). Comparison of data for the two soil depths indicated that fungal taxa from Nectriaceae and Tremellaceae families were the most discriminating taxa (Two-sided Welch’s test, *P* < 0.05); Nectriaceae being predominant in the surface 0–5 cm soils and Tremellaceae vice versa. Results from Bio-Env test showed significant links between community composition and soil properties including pH, Exchangeable Cu, DTPA-extractable Mn, Exch. Ca, Colwell P, % sand (BIO-Env test: ρ = 0.58, *P* = 0.01) (Figure 7). Additionally, application of DistLM analysis (Distance based linear models) indicated that soil variables including pH, Exchangeable K and Exchangeable Ca, ECEC, % sand and Boron levels contributed ∼10% each (significant at *P* = 0.001) to the zone based variation.

### Effect of Mulching

Comparison of the bacterial composition in the mulched vs. no-mulch soils from the Medium and High rotundone zones using distance based redundancy analysis (dbRDA) showed no significant variation (ANOSIM *R* = 0.137, *P* = 0.18) with no changes to the rotundone zone based dissimilarity (Supplementary Figure S2). However, significant mulching effects (Two-sided Welch’s test, *P* < 0.05) could be seen in the variation in relative abundances of bacterial genera Sphingomonadaceae_g, GP6, Betaproteobacteria_g, Rhodocyclaceae_g, GP16, Burkholderiales_g, Sphingomonas, Rhodospirillaceae_g, Thermosporothrix and GP7.

Unlike the bacteria, there was a significant variation in fungal community composition from mulching (ANOSIM *R* = 0.225, *P* = 0.03; PERMANOVA CV 14.7, *P* = 0.001) (Supplementary Figure S2). However, these changes did not influence or mask the rotundone zone based variation (Supplementary Figures S2B,D). Fungal taxa associated with the family Nectriaceae were predominant in the mulched soils from both the Medium and High rotundone zones, whereas taxa belonging to the families Tremellaceae and Pezizomycotina_incertae_sedis were higher in the no-mulch soils.

### Microbial Networks

#### Bacteria

**FIGURE 8:**
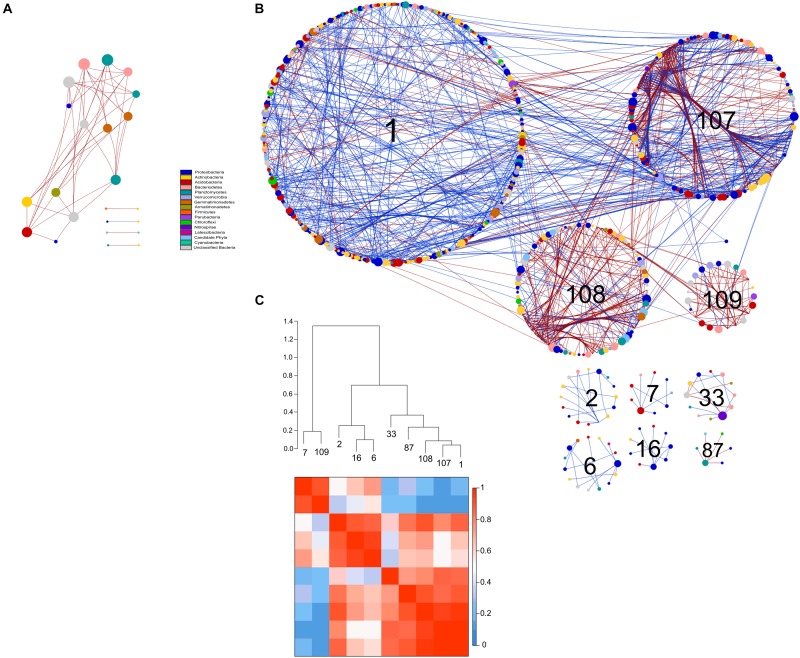
Comparison of rotundone-Low **(A)** and rotundone-High without mulch **(B)** bacterial networks. Circles represent nodes whose size indicates connectivity, node color represents taxonomy at the phyla level. Edges indicate co-occurrence between nodes colored either blue for positive or red for negative. Each circular grouping is a module. Numbers within modules correspond to numbers indicated in the hierarchical clustering. **(C)** Hierarchical clustering based on Pearson correlations among module-eigengenes and a heatmap of module eigengenes of the rotundone-High without mulch network.

Results from bacterial co-occurrence network analysis revealed large differences between rotundone zones. For example, 16S-high and 16S-highNM networks contained more nodes and links compared to the 16S-low network (Table 2A). In both 16S-high and 16S-highNM networks, the direction of the interactions were more evenly split between positive (56–58%) and negative (42–44%) links. In the 16S-low network, nearly all interactions were in the negative with the exception of one positive interaction between a Proteobacteria (Rhizobiales) and an Actinobacteria (Gaiellaceae) (Figure 8A). The modularity index of the 16S-high and 16S-highNM networks were greater than 0.4, indicating that these networks were modular in structure (Table 2A) with 13 and 10 modules that contained at least 5 nodes in the 16S-high and 16S-highNM networks (Supplementary Figure S4 and Figure 8B). The value of the coefficient of determination (*R*^2^) of power law was 0.93 in the rotundone High networks, indicating scale-free network characteristics (Table 2A). In contrast, the modularity index were below the threshold and the value of the *R*^2^ did not fit the power law model for the 16S-low network, which may indicate that this network does not exhibit scale-free characteristics and does not have a modular structure (Figure 8A and Table 2A). Additionally, there were little differences between the 16S-low network and the randomly generated networks using identical numbers of nodes and edges (Table 2 and Supplementary Table S5).

**TABLE 2:** Topological properties of molecular ecological networks for soil bacterial **(A)** and fungal **(B)** communities.

**(A)**			
**Network property**	**Rotundone-High**	**Rotundone-HighNM**	**Rotundone-Low**
RMT threshold	0.870	0.920	0.980
Modularity	0.572	0.603	0.156
Total nodes	683	916	23
Total links	1313	1754	50
*R*^2^ of power-law	0.933	0.931	0.004
Average degree (avgK)	3.845	3.830	4.348
Ave. clustering coefficient (avgCC)	0.065	0.055	0
Harmonic geodesic distance (HD)	4.5	4.967	1.4

**(B)**			

RMT threshold	0.700	0.770	0.810
Modularity	0.518	0.522	0.576
Total nodes	135	118	181
Total links	224	207	266
R^2^ of power-law	0.97	0.8	0.904
Average degree (avgK)	3.319	3.508	2.939
Average clustering coefficient (avgCC)	0.042	0.099	0.074
Harmonic geodesic distance (HD)	3.438	3.384	4.079

The modules in the 16S-high and 16S-highNM networks generally grouped to three clusters (Figures 8B,C and Supplementary Figure S4). The three largest modules (#1, #107, and #108) in the largest cluster in the 16S-highNM network contained the majority of the positive interaction in the network (Figures 8B,C). The second cluster in the 16S-highNM network contained modules #2, #6, and #16; all interactions in this cluster were positive, although there were no interactions between modules (Figure 8B). In both the clusters, Proteobacteria and Actinobacteria were the most abundant bacterial phyla. Whereas, Acidobacteria were the most abundant phyla in the modules #7 and #109, third cluster in the 16S-highNM network. Mulching effects appear to expand the network, although the number of nodes and links were greater in the 16S-highNM network. The 16S-high network contained more modules compared to the 16S-NM network, but the number of nodes per module were more consistent (Supplementary Figure S4). The modules of the 16S-high network generally clustered to three groups, except module #46 which was not strongly correlated with other modules in the network (Supplementary Figures S4B,D). The correlations between module-based eigengenes and environmental variables can be used to detect the modules’ responses to environmental changes. The 16S-low network lacked the modularity to run the module-eigengene analysis. In the 16S-high and 16S-highNM networks, the coefficients and significances are shown in a heatmap (Supplementary Figure S6).

All nodes of the 16S-low network were classified as peripheral nodes. In the 16S-high network, 2 network hubs, 16 module hubs and 56 connectors were identified (Supplementary Figure S8 and Supplementary Table S7). Approximately half of the module hubs were identified as Rhizobiales or Rhodospirillales belonging to the class Alphaproteobacteria. The module hubs also included the Acidobacteria, Actinobacteria, Armatimonadetes, and Planctomycetes. Among the connectors, the majority of nodes were classified as Proteobacteria and Acidobacteria. However, the Acidobacterial groups classified as connectors differed from the module hub Acidobacterial groups (Supplementary Table S7). Overall, the 16S-highNM network, contained a lower number of connectors but increased the number of module hubs and were more diverse although Proteobacteria and Acidobacteria remained the most abundant taxa in each topological category.

#### Fungi

**FIGURE 9:**
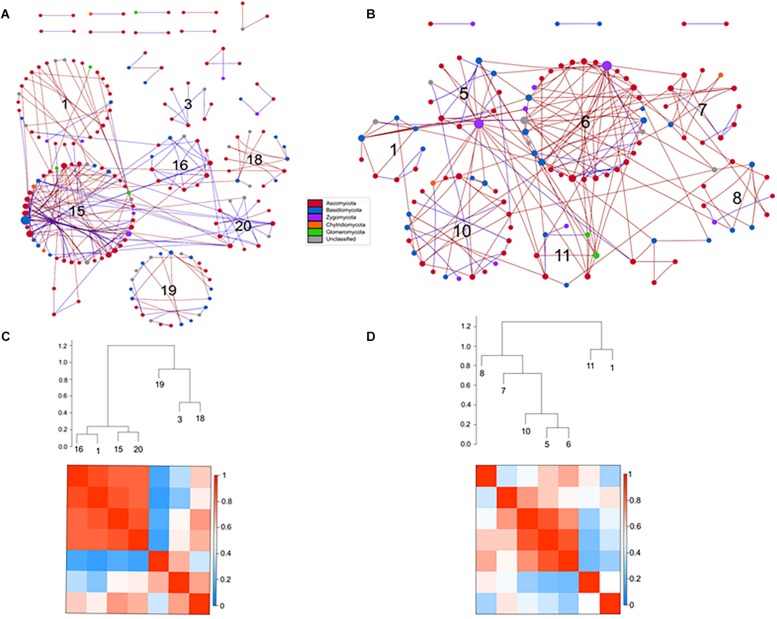
Comparison of rotundone-Low **(A)** and rotundone-High without mulch **(B)** ITS networks. Circles represent nodes whose size indicates connectivity, node color represents taxonomy at the phyla level. Edges indicate co-occurrence between nodes colored either blue for positive or red for negative. Each circular grouping is a module. Numbers within modules correspond to numbers indicated in the hierarchical clustering. Hierarchical clustering based on Pearson correlations among module-eigengenes and a heatmap of module eigengenes of the **(C)** rotundone-Low network **(D)** rotundone-High without mulch network.

Compared to the bacterial 16S networks, the fungal ITS networks were less complex and contained fewer nodes and links (Table 2B). All fungal networks exhibited scale-free characteristics indicated by the value of the *R*^2^ of power law and had a modularity index greater than 0.4. The resulting ITS-high network contained eight modules and both ITS-low and ITS-highNM networks contained 7 modules. The direction of the interactions in the ITS-low network were evenly split between positive (131; 49%) and negative (133; 51%) interactions (Figure 9A). Whereas, the ITS-high and ITS-highNM networks contained a higher number of negative interactions (76%; 171 and 157 links in ITS-high and ITS-highNM, respectively) than positive interactions (28%; 50 and 53 links in ITS-high and ITS-highNM, respectively) (Figure 9B and Supplementary Figure S5).

The modules of the ITS-low network generally grouped to two clusters with modules #1, #16, #15, and #20 in the first cluster strongly correlated (Figure 9C). The dominant fungal taxa in the first cluster were the Pezizomycotina belonging to the phylum Ascomycota which composed 64–80% of each module. In the second cluster, module #3 was primarily composed of Ascomycota, in contrast modules #18 and #19 were composed of Ascomycota (29–43%) and Basidiomycota (36–46%). In the ITS-high network, one cluster of modules (#12, #13, #9, #7, and #11) were highly correlated with Pezizomycotina (44–78%) and Agaricomycota (11-14%) as the dominant taxa (Supplementary Figure S5D). Similarly, the ITS-highNM network contained one cluster module (modules #5, #6, #10) which were strongly correlated and the dominant taxa in the cluster were the Pezizomycotina (63%) and an unclassified Basidiomycota (18–25% of each module) (Figure 9D). The correlations between module-based eigengenes and environmental variables to detect the fungal modules’ responses to environmental changes were identified (Supplementary Figure S7).

Classification of the topological roles of each node in the networks identified four module hubs and three connectors in the ITS-low network (Supplementary Table S6 and Supplementary Figure S8). Three of the four module hubs were identified as Basidiomycota and two of three connectors were identified as Ascomycota (Supplementary Table S8). In both the ITS-high and ITS-highNM networks, 1 network hub, 3 module hubs, and 9 and 10 connectors were identified. Overall, connectors in the High rotundone samples were identified as Ascomycota, mulching did not have an effect on the identity of the connectors. However, mulching led to the Zygomycota module hubs nodes being replaced by Ascomycota (Supplementary Table S8).

### Catabolic Diversity and Activity

**FIGURE 10:**
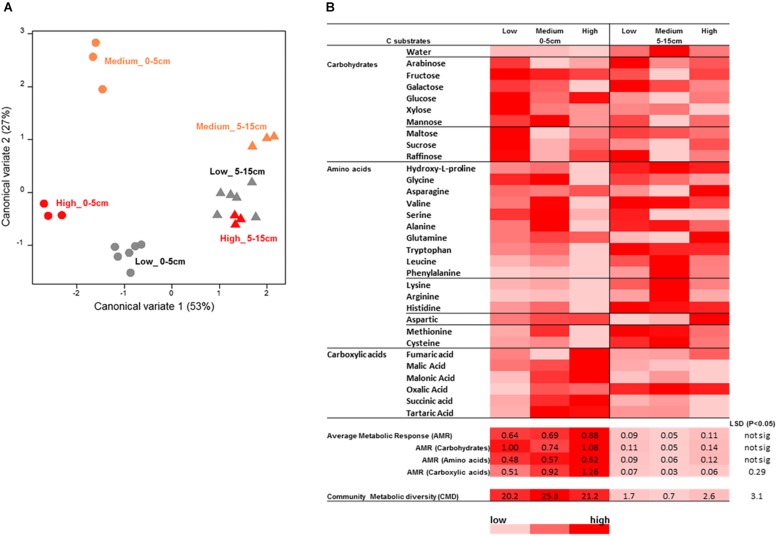
Microbial catabolic profiling analysis results for the surface soil samples from the rotundone-zone soil samples. **(A)** Canonical variate analysis (CVA) plot showing the dissimilarity in the catabolic diversity of soil microbial communities, **(B)** heat map of the AWCD showing differences in substrate use efficiency for the various C-substrates by the soil microbial communities.

Analysis of soil microbial catabolic diversity as assessed by MicroResp^®^ (community level physiological profiling, CLPP) method showed significant differences, tested using the zone × depth model, between the rotundone zones. However, these were only seen in the surface 0–5 cm soil whereas the 5–15 cm samples grouped together (Figures 10A,B). There was a significant soil depth based difference in microbial catabolic diversity and average metabolic response (AMR). Soils from the Low rotundone zone generally exhibited lowest microbial average metabolic response (AMR; 0.69 ± 0.04) and community metabolic diversity (CMD; 20 ± 1) compared to those from the High rotundone zone (AMR = 0.87 ± 0.05 and CMD = 27 ± 0.36), especially in the surface 0–5 cm (Figure 10). The average AMR value for carboxylic acid group of substrates was significantly higher in the High and Medium rotundone zone soils compared to that in the Low rotundone soils. But such differences were not seen for the carbohydrates and amino acid group of substrates. Principle component analysis (PCA) of mulching effects showed that 57% of variance in catabolic profiling data was explained by the first two PCA axes indicating the significant variation in microbial activity and response to C substrates in soils from the Medium and High rotundone zones and both depths (Supplementary Figure S3A). Mulching generally increased microbial activity responses to C substrate addition in both the zones, but the grouping remained similar between the zones and depths (Supplementary Figure S3B). However, mulching significantly increased AMR (67%) and CMD (27%) in the High-rotundone zone soils only (Supplementary Figure S3B). Mulching specifically increased AMR for carbohydrate group of substrates in the surface 0–5 cm soils (Supplementary Figure S3).

## Discussion

The role of vineyard soil microbial communities and plant–microbe interactions influencing the grape berry microbiome has received increasing attention based on the findings that berries harbor diverse bacterial communities mainly originating from the vineyard soil environment (Zarraonaindia et al., 2015; Mezzasalma et al., 2017). Overall, our findings show distinct differences in bacterial and fungal community structures in surface soils between vineyard zones that vary in the concentration of grape berry rotundone, a key aroma compound responsible for the much sought after ‘peppery’ character that is distinctive of premium cool climate Shiraz wines. Previous research had shown a temporally stable pattern of within-vineyard variation in this grape-derived flavor and aroma compound across a 40-fold range of variation in the annual mean rotundone concentration (Bramley et al., 2017; Figure 1). This temporal stability of concentration differences in the grape metabolite rotundone could be confirmed for the time the soil samples were taken in 2017. This provided essential support for the hypothesis that within-vineyard variation in rotundone is associated with differences in soil properties and/or topography and suggested the possibility that soil biological factors may be related to the patterns of rotundone variation (Bramley et al., 2017). While the present results do not infer a cause and effect relationship between grape and wine pepperiness and soil microbial composition, they do lend further weight to a possible role for the microbiome in contributing to wine *terroir* and to the need for further research to explore functional consequences from differences in the microbial composition and associated differences in wine characteristics (*terroir* or rotundone concentrations) of vineyard zones.

Next generation sequencing analysis of soil microbial communities has revealed an overwhelming diversity of bacterial communities, even in managed agricultural and viticultural soils which might be expected to be degraded by comparison to their native state (Fierer and Jackson, 2006; Zarraonaindia et al., 2015; Bissett et al., 2016). Our results from an Australian vineyard soil indicate that Proteobacteria, Actinobacteria, Acidobacteria, Gemmatimonadetes, and Verrucomicrobia are the top 5 most abundant groups accounting for 84% of the total bacterial community. The top 5 most abundant phyla in vineyard soils outside Australia vary only slightly and include Proteobacteria, Bacteriodetes, Acidobacteria, Verrucomicrobia and Planctomycetes, (Morgan et al., 2017; Novello et al., 2017). Similarly, members of bacterial Orders Enterobacteriales, Pseudomonadales, Bacillales, and Rhodospirillales are considered ‘common microbiome’ of vineyard soils which seem to not respond to pedological and environmental conditions (Mezzasalma et al., 2017). *Gaiella* was also the dominant genus of Actinobacteria in the rhizosphere microbiota in Piedmont, Italy (Novello et al., 2017).

Many factors, including edaphic and environmental factors along with management are involved in determining the soil microbial (bacterial and fungal) composition and activity. Host plant phenology and seasonal variables can also affect the composition and abundance of active microbial communities in a vineyard soil (Gilbert et al., 2014). In this study, soil organic C, the chief source of energy for the heterotrophic microorganisms did not show significant relationships on either the abundance or diversity of bacteria and fungi in the non-mulched soils, suggesting that soil C variation may not driving differences in microbial diversity within this vineyard. In our grapevine soils we found that soil pH, exchangeable Ca, Mn, Na, and sand content were the major predictors of bacterial composition in the surface soils. Similarly, variation in the fungal composition was influenced by the above factors and exchangeable Cu and plant-available Colwell P. While biogeography in the berry microbial community structure has been reported for Chardonnay and Cabernet Sauvignon musts in California (Bokulich et al., 2014), edaphic factors within vineyards played a greater role in determining the bacterial and fungal communities in vineyards in Long Island (United States; Zarraonaindia et al., 2015), highlighting their importance for soils with homogeneous climatic conditions.

Much of the previous work on wine *terroir* has involved research conducted at regional scale, yet as Bramley (2017) has suggested, understanding of *terroir* will remain elusive unless it is investigated at much finer spatial scales, such as the within-vineyard scale used in the present study. Our observation that within-vineyard variation in soil microorganisms, in terms of the relative distribution of specific members of bacterial and fungal community, correlated to differences in rotundone concentration could have implications for management. However, evidence of microbial community impacts on management only currently exist with respect to the grape and must microbiomes; the influence, direct or indirect, of the soil microbiome is not clearly established (Bokulich et al., 2014; Zarraonaindia et al., 2015). Of interest here is that an established *terroir* effect in terms of wine pepperiness, aligns with differences in the soil microbiome at the within-vineyard scale. Bramley et al. (2017) have explained how, depending on seasonal conditions, the pepperiness of final wines might be altered through decisions taken in the vineyard, such as selective harvesting. Davies et al. (2015) have suggested that rotundone might similarly be manipulated through the targeted use of ripening modulators, whilst Geffroy et al. (2014) have suggested targeted pruning as a means of manipulating rotundone levels. Manipulation of soil microbial communities through incorporation of management practices considering the observed predictors of microbial community, such as specific fertilizer application, could also be an avenue worthy of exploration but requires a better understanding of the functional consequences of these distinct microbial communities as opposed solely to their composition as studied here.

The biogeography or local heterogeneity in microbial composition infers location-specificity of the soil microbiome (Mezzasalma et al., 2017). Our results show that distinct parts of the soil microbiome, that is, 514 and 454 OTUs of bacteria and fungi, were specifically found in the High rotundone zone soils. There were also bacterial and fungal OTUs that were only seen in the Low and Medium rotundone soils. Locality based microbiome composition, expressed as unique microbial traits in the must and grapes in the early stages of fermentation has been reported before, e.g., representatives of Enterobacteriales, Pasteurella, Rhodospirillales (Bokulich et al., 2014; Mezzasalma et al., 2017). The functional importance of such members in determining wine quality or *terroir* is not known.

Although the abundance of bacteria was lower in the High and Medium rotundone soils, they exhibited higher diversity, suggesting that the plants in the High rotundone soils had a more diverse soil reservoir to access for beneficial interactions. Similarly, the role of lower diversity and abundance of soil fungi in the higher rotundone soils is not clear. The effect of lower abundance of soil fungal community could be through fewer pathogenic species. As the information from the amplicon sequencing conducted in this study does not allow identification of fungi to species level it is difficult to determine the proportion of the various functional groups and specific pathogenic species (Nguyen et al., 2016). A number of Glomeromycota fungi form arbuscular mycorrhizas with vine plants and are known to provide beneficial functions. However, we found no significant differences in the proportion of Glomeromycota in the different rotundone zone soils.

Network analysis of microbial community data provides information that delineates the community structure about potential linkages and interactions between its members (Zhou et al., 2010). While the network analysis can help identify keystone taxa based on statistical correlations by assuming positive and negative edges represent mutual co-presence and exclusion, further experimental validation is required to confirm mechanistic linkages. The bacterial community network in the High rotundone zone soils was highly structured, both in the no-mulch and mulched soils, compared to the Low-rotundone zones, as evidenced by the network properties such as modularity, number of nodes and links and clustering co-efficient which were generally high in the High rotundone soils. It is suggested that in a community network, the nodes (members of the community) that are highly connected with other members and the connectors (those linking modules) have important roles in maintaining the network integrity; that is, they serve as putative keystone taxa that provide stability to the microbial community (Olesen et al., 2007). Such putative keystone taxa identified for the bacterial community in the High rotundone zone soils were not found in the Low rotundone zone soils which could be the reason for the poor network structure in these soils. In the case of the fungal community, the general network complexity differences were small, the keystone taxa were different between the different rotundone zone soils. Overall, the high level of organization along with higher diversity, especially for the bacterial community in the High rotundone zone soils would provide the vine plant with a stable microbial reservoir across varied seasonal environmental conditions. Higher diversity of rhizosphere recruited microbiome has been suggested to help plant withstand biotic and abiotic stresses through regulation of gene expression for plant immune responses, e.g., alterations to terpenoid synthesis, ethylene function related plant response (Glick, 2014; Schuman and Baldwin, 2018). Some of the putative keystone taxa derived from network hubs in the High rotundone zone soils, connectors such as members of Chitinophagaceae, Nitrospira, Rhodospirillaceae, and Gaiellaceae, Rhizobiales are known to contribute to plant growth promotion, induction of genes such as related to ethylene production and tolerance to abiotic stress (*acd*S), nutrient (N) mineralization, and disease control properties (Compant et al., 2005; Glick, 2014; Backer et al., 2018). The functional significance of the Acidobacteria groups, i.e., GP4, GP7 etc., is not known. In this study, mulch appeared to affect which Acidobacterial subgroups act as connectors and module hubs in the bacterial networks; Naether et al. (2012) indicated that for detecting novel interrelations of environmental parameters with Acidobacteria, individual populations within subgroups have to be considered.

It has been suggested that vineyard soil acts as a reservoir of bacteria during the vegetative period ready to colonize the above-ground endophyte communities and the external aerial part of the plants including berries (Zarraonaindia et al., 2015). Additionally, rain splash, wind erosion and insects would also lead the transfer of soil bacteria to epiphytic microbial communities. Although geographical variation is seen in vineyard soil microbiomes, it may not always directly reflect in the berry microbiome (Martins et al., 2012; Zarraonaindia et al., 2015; Chou et al., 2018). A diverse and responsive soil microbial community in the High rotundone zone gives the plants a more diverse and responsive community to exploit for beneficial functions such as tolerance to biotic and abiotic stresses, nutrient availability etc. Also, well-connected microbial community networks are stable through the different seasons and a stable core microbiome of vineyards could be one of the basic biotic elements that would influence above-ground microbiome including berry microbiome (Pinto et al., 2014) contributing to the stable patterns of spatial variation in rotundone concentration (Bramley et al., 2017).

Results from the activity based assay measuring multiple C substrate utilization capacity provided an indication of the responsiveness of the different members of microbial community to management practices that add external C sources such as mulch, especially in the High rotundone zone soils (Garland, 1997). Our findings showed significant increases in the community metabolic response and diversity from mulching and clear and distinct differences in the CLPP based on rotundone zone and soil depth. However, the microbial community showed no significant and systematic change due to mulching in the two rotundone zones that masked the zone-based variation, although significant changes were seen in some groups.

Soil and management factors such as ground cover management, organic manure, herbicide and fungicide application and cultivation have been shown to significantly influence bacterial and fungal communities in vineyard soils (Dell’Amico et al., 2008; Martins et al., 2012; Burns et al., 2016; Chou et al., 2018; Vukicevich et al., 2018). In this study, soils from the mulched areas of the Medium and High rotundone zone showed significant changes in some of the fungal groups, e.g., Nectriaceae, Tremellaceae, and Pezizomycotina_incertae_sedis. Some of the members of the above groups are known decomposers and so would have responded to addition of an organic C source such as mulch, but these did not modify/mask the rotundone zone based differences in overall fungal community structure. It is known that soil C resources generally drive the diversity of soil microbial communities in agricultural soils including vineyard soils (Burns et al., 2016; Morgan et al., 2017), yet we only found a significant increase in the phylogenetic diversity due to mulching with fungi, not with bacteria (Supplementary Table S4). Overall, these results suggest that either the mulching induced changes in microbial community structure have no direct link to the microbiome variation between the different rotundone zones or that the effect of mulching was yet to be expressed, although given that mulch was applied 7–8 months prior to soil sampling during the wetter part of the year, this lack of mulch expression seems unlikely. Additionally, it has also been reported that while under-vine management can cause shifts in the soil microbiome, these may not necessarily be accompanied by changes in fruit-associated microbial composition even though aerial organ-associated bacteria likely originated from soil (Zarraonaindia et al., 2015; Burns et al., 2016; Chou et al., 2018).

Overall, this descriptive genomics based finding needs to be extended to understand the functional importance of the specific members of microbial community varying between zones. The changes in phyllosphere induced by seasonal growth of vine plants and berries makes their interaction with the microbiome more complex but the soil microbiome is considered as a reservoir for the above-ground foliage and in turn influence berry production and quality. However, if the phyllosphere and berry microbiome in the different rotundone zones follow the same trend seen in the soil microbiome then it could have some role in rotundone formation. Additionally, rotundone is thought to form in the skins of berries late in the season, just before harvest (Geffroy et al., 2014), a factor which raises questions as to the timing of any soil microbiome effect. Therefore, future research should investigate the direct links between soil microbiome, the microbiome in wine ferments and ultimately, wine quality. Furthermore, manipulation of soil microbial communities could also be an avenue worthy of exploration albeit following a better understanding of the functional consequences of these distinct microbial communities.

## Conclusion

A better understanding of the environmental, genetic and biological factors that drive or contribute to the *terroir* of wines is critical for the development of targeted vineyard management strategies. Our results have clearly demonstrated, as a first report of this type, that distinct differences in soil bacterial and fungal community composition and structure in different zones within the same vineyard are associated with different propensities for grape berry rotundone concentration. The High rotundone zone soils exhibited higher diversity of bacteria but lower diversity of fungi compared to the soils in the Low rotundone zone. The dissimilarity in the microbial community structure between the rotundone zones is concentrated in a few taxa/groups of both bacteria and fungi. Also, the bacterial community co-occurrence network in the High rotundone zone soil exhibited a well-connected network by comparison with the Low rotundone zone soil. Although mulching in a part of the vineyard caused changes in bacterial and fungal composition and overall microbial catabolic diversity and activity, its effects did not mask the between-zone variation. Overall, these results have potentially important implications for grape and wine research, viticultural management and grape production, and the understanding of wine *terroir*. They also lend weight to the notion that such understanding will depend on research conducted at finer spatial scales.

## Data Availability

The datasets for the 16S rRNA (bacteria) and ITS region (fungi) sequences and associated soil metadata generated and analyzed for this study can be found at the CSIRO data access portal https://doi.org/10.25919/5c73199923fb1 (Gupta and Greenfield, 2019).

## Author Contributions

VG contributed to all aspects of the study. RB was involved in selecting vines within zones for sampling and rotundone concentration mapping. PG conducted the bioinformatic analysis. JY was involved with network analysis. MH was involved with experimental design and rotundone concentration measurement. All authors contributed to the preparation of the manuscript.

## Conflict of Interest Statement

The authors declare that the research was conducted in the absence of any commercial or financial relationships that could be construed as a potential conflict of interest.

## Footnotes

1 www.apal.com.au

2 www.leco.com

3 www.qiagen.com

4 www.qiagen.com.au

5 https://support.illumina.com

6 www.agrf.org.au

7 https://rdp.cme.msu.edu/

8 http://ieg4.rccc.ou.edu/MENA/login.cgi
