# Supplementary material for: Vineyard Soil Microbiome Composition Related to Rotundone Concentration in Australian Cool Climate ‘Peppery’ Shiraz Grapes
Source: Front Microbiol. 2019 Jul 16;10:1607. doi: 10.3389/fmicb.2019.01607 (PMC6646731; doi:10.3389/fmicb.2019.01607)
Supplement: Supplementary file 1 [file Data_Sheet_1.PDF]

Supplementary

Table S1. Detailed results for physical and chemical properties for soils from different depths, mulching treatments and zones.

Zone	Low		Medium				High										ANOVA (LSD P<0.05)			
Mulch	No Mulch		Mulch		No Mulch		Mulch		No Mulch		Depth		Mulch		No Mulch		Depth	Mulch x Depth		
Depth (cm)	0-5	5-15	0-5	5-15	0-5	5-15	0-5	5-15	0-5	5-15	0-5	5-15	0-5	5-15	0-5	5-15		Mulch	Depth	M x D
pH(Water)	7.2	6.7	7.1	6.8	7.0	6.1	7.2	6.8	7.4	6.6	7.2	6.6	7.2	6.8	7.2	6.3	0.17	NS	0.209	
Organic Carbon (%)	2.35	1.63	3.34	2.10	2.22	1.60	3.00	1.12	1.94	1.15	2.6	1.5	3.2	1.6	2.1	1.4	0.31	NS	0.423	
TN (µg/g)	5.9	11.3	5.3	6.4	2.3	6.0	6.3	5.2	4.6	4.9	4.9	6.8	5.8	5.8	3.4	5.5	0.36	NS	0.459	0.562
DOC (µg/g)	26.5	25.4	60.0	37.6	68.1	25.8	21.6	9.8	38.6	8.1	43.0	21.3	40.8	23.7	53.4	17.0	12.10	NS	12.1	NS
MinN (µg/g)	3.14	9.54	1.66	0.74	0.01	5.68	3.86	3.97	2.46	4.66	2.2	4.9	2.8	2.4	1.2	5.2	2.26	NS	NS	NS
Colwell P (µg/g)	16.4	6.3	16.0	10.0	21.8	14.0	26.4	15.8	31.2	34.2	22.4	16.1	21.2	12.9	26.5	24.1	5.41	87.3	NS	NS
Colwell K (µg/g)	220	122	457	218	227	96	229	136	104	52	247.4	124.7	343.0	177.0	165.3	73.9	45.60	NS	58	NS
KCL Sulfur (µg/g)	7.20	7.83	7.50	8.00	9.18	10.72	6.62	5.02	5.54	5.36	7.2	7.4	7.1	6.5	7.4	8.0	NS	NS	NS	NS
DTPA-Cu (mg/kg)	9.76	4.08	13.65	4.94	7.82	2.27	21.42	9.90	11.32	5.46	12.8	5.3	17.5	7.4	9.6	3.9	2.67	NS	3.45	NS
DTPA-Zn (mg/kg)	3.33	0.94	8.63	2.05	3.27	0.71	12.78	2.26	6.94	1.73	7.0	1.5	10.7	2.2	5.1	1.2	1.62	NS	2.26	NS
DTPA-Mn (mg/kg)	2.19	1.35	4.89	2.35	2.25	1.50	2.96	1.19	1.67	0.86	2.8	1.5	3.9	1.8	2.0	1.2	0.46	0.765	0.511	0.625
DTPA-Fe (mg/kg)	24.4	36.8	39.6	35.9	25.6	48.2	24.8	29.4	18.2	31.8	26.5	36.4	32.2	32.7	21.9	40.0	6.97	0.511	7.59	NS
Exchangeble CEC	10.54	7.36	13.91	9.38	9.56	5.65	11.79	5.07	9.34	4.49	11.0	6.4	12.8	7.2	9.4	5.1	1.07	0.625	1.303	NS
EC 1:5	0.046	0.054	0.057	0.045	0.049	0.055	0.055	0.035	0.043	0.033	0.0	0.0	0.1	0.0	0.0	0.0	NS	NS	NS	NS
Boron	0.67	0.31	0.88	0.43	0.49	0.24	0.78	0.22	0.56	0.22	0.7	0.3	0.8	0.3	0.5	0.2	0.09	NS	NS	NS
Exch K	0.43	0.21	0.93	0.39	0.36	0.11	0.44	0.25	0.18	0.08	0.5	0.2	0.7	0.3	0.3	0.1	0.10	NS	NS	NS
Exch Ca	8.33	5.69	10.53	7.19	7.52	3.89	9.16	3.76	7.76	3.47	8.7	4.8	9.8	5.5	7.6	3.7	0.83	NS	0.952	NS
Exch Mg	1.67	1.35	2.38	1.69	1.47	1.31	2.08	0.96	1.28	0.84	1.8	1.2	2.2	1.3	1.4	1.1	0.22	NS	0.294	0.36
Exch Na	0.11	0.11	0.06	0.10	0.21	0.24	0.11	0.10	0.13	0.10	0.1	0.1	0.1	0.1	0.2	0.2	NS	0.718	NS	NS
Ca:Mg ratio	5.01	4.27	4.36	4.21	5.16	3.14	4.63	3.98	6.74	4.16	5.2	4.0	4.5	4.1	5.9	3.7	0.49	NS	NS	NS
Clay (%)	4.9	5.7	5.3	5.6	5.2	8.8	2.4	2.8	4.6	4.4	4.5	5.5	3.8	4.2	4.9	6.6	NS	NS	NS	NS
Sand (%)	43.7	41.6	42.9	40.9	39.0	36.1	62.1	56.4	62.4	63.5	50.0	47.7	52.5	48.6	50.7	49.8	NS	NS	NS	NS
Silt (%)	51.5	52.7	51.8	53.6	55.8	55.1	35.5	40.8	33.0	32.2	45.5	46.9	43.7	47.2	44.4	43.7	NS	NS	NS	NS

Note: NS – not significant

Table S2. Sequences and OTUs obtained for different soil zones.

Property	Bacteria (16S)	Fungi (ITS)
Total sequences	2,181623	4,327,722
Ave. seq/sample	41,954	83,225
Total OTUs	13,283	3,783
Ave. OTU/sample	3,443	482
0-15 cm	3571 ±72	547 ±28
5-15 cm	3315 ±96	417 ±19
No-mulch	3465 ±74	476 ±24
Mulch	3371 ±107	502 ±24

Table S3. PERMANOVA analysis. Statistical comparisons of the 16S rRNA and ITS region dataset for the effects of zone, depth and mulching

Dataset	Factor	ANOSIM		PERMANOVA	
		Global R	P	CV	P
16S rRNA	Zone	0.886	0.001	17.3%	0.001
	Depth	0.699	0.001	22.6%	0.001
	Zone without depth	0.414	0.001	16.6%	0.001
	Mulch	0.251	0.40	3.9%	0.032
ITS region	Zone	0.565	0.001	17.2%	0.001
	Depth	0.683	0.001	20.3%	0.001
	Zone without depth	0.420	0.001	16.7%	0.001
	Mulch	0.318	0.02	14.7%	0.001

CV = Components of variation

Table S4. Diversity measures of bacterial and fungal communities (average ± stderr) as influenced by the rotundone zone, soil depth and mulching.

			Bacteria				Fungi			
**Zones**	**Mulch**	**Depth (cm)**	**OTUs**	**Margalef's species richness (d)**	**Pilou's evennes (j)**	**Shannon index (H)**	**OTUs**	**Margalef's species richness (d)**	**Pilou's evennes (j)**	**Shannon index (H)**
**Low**	**No Mulch**	**0-5**	3430 ±105	910 ±25	0.968 ±0.001	7.880 ±0.030	627	230 ±6	0.925 ±0.001	5.955 ±0.036
		**5-15**	3246 ±131	869 ±31	0.966 ±0.001	7.808 ±0.041	457	182 ±8	0.914 ±0.003	5.591 ±0.057
**Medium**	**Mulch**	**0-5**	3514 ± 87	924 ±21	0.970 ±0.001	7.921 ±0.026	480	187 ±12	0.918 ±0.007	5.667 ±0.052
		**5-15**	3439 ±229	913 ±53	0.967 ±0.001	7.873 ±0.068	377	149 ±8	0.922 ±0.000	5.463 ±0.061
	**No Mulch**	**0-5**	3512 ±141	929 ±32	0.970 ±0.000	7.913 ±0.038	416	163 ±11	0.922 ±0.005	5.547 ±0.073
		**5-15**	2959 ±271	812 ±60	0.961 ±0.003	7.667 ±0.106	301	123 ±10	0.925 ±0.003	5.264 ±0.079
**High**	**Mulch**	**0-5**	3191 ±252	851 ±59	0.970 ±0.001	7.817 ±0.082	502	192 ±14	0.920 ±0.008	5.714 ±0.104
		**5-15**	3339 ±312	887 ±74	0.969 ±0.001	7.853 ±0.097	384	153 ±17	0.919 ±0.006	5.449 ±0.198
	**No Mulch**	**0-5**	3961 ± 69	1030 ±16	0.971 ±0.000	8.040 ±0.019	342	143 ±11	0.915 ±0.008	5.322 ±0.080
		**5-15**	3586 ±184	946 ±41	0.968 ±0.001	7.916 ±0.062	289	128 ±6	0.905 ±0.008	5.120 ±0.070
	**Zones**									
	Low		3338	886	0.9673	7.837	542	206	0.9197	5.773
	Medium		3356	871	0.9654	7.790	358	143	0.9233	5.4054
	High		3519	988	0.9693	7.978	316	135	0.9099	5.2206
	LSD (P<0.05)		*337*	*77*	*0.0021*	*0.113*	*47*	*15.7*	*NS*	*0.1225*
	**Depths**									
	0 - 5 cm		3635	956	0.9695	7.942	462	179	0.9207	5.6077
	5 - 15 cm		3264	876	0.9652	7.797	349	144	0.9146	5.3249
	LSD (P<0.05)		*269*	*62*	*0.0018*	*0.091*	*37*	*13*	*0.0057*	*0.0965*
	**Mulching**									
	Mulch		3371	894	0.9691	7.866	436	170	0.9198	5.573
	No-Mulch		3505	929	0.9673	7.883	337	139	0.9166	5.313
	LSD (P<0.05)		*NS*	*NS*	*NS*	*NS*	*87*	*29.4*	*NS*	*0.2303*

Note: Averages for the zones and depths are for No-mulch treatment only.

Table S5. Additional topological properties of molecular ecological networks for soil bacterial communities.

Network property	Rotundone-High	Rotundone-HighNM	Rotundone-Low
Average path distance (GD)	5.593	6.087	1.633
Geodesic efficiency (E)	0.222	0.201	0.714
Harmonic geodesic distance (HD)	4.51	4.967	1.4
Maximal degree	42	47	8
Nodes with max degree	OTU_1787	OTU_134	OTU_3608;OTU_4543;OTU_9457
Centralization of degree (CD)	0.056	0.047	0.182
Maximal betweenness	31351.074	31954.525	8.827
Nodes with max betweenness	OTU_1787	OTU_134	OTU_1099;OTU_920
Centralization of betweenness (CB)	0.131	0.074	0.026
Maximal stress centrality	731067	10298217	67
Nodes with max stress centrality	OTU_1787	OTU_134	OTU_1099;OTU_920
Centralization of stress centrality (CS)	3.055	24.207	0.192
Maximal eigenvector centrality	0.289	0.242	0.288
Nodes with max eigenvector centrality	OTU_1787	OTU_134	OTU_1099;OTU_920; OTU_3608;OTU_4543;OTU_9457
Centralization of eigenvector centrality (CE)	0.273	0.232	0.13
Density (D)	0.006	0.004	0.198
Reciprocity	1	1	1
Transitivity (Trans)	0.08	0.067	0
Connectedness (Con)	0.616	0.439	0.431
Efficiency	0.993	0.993	0.59
Hierarchy	0	0	0
Lubeness	1	1	1

Table S6. Additional topological properties of molecular ecological networks for soil fungal communities.

Network property	Rotundone-High	Rotundone-HighNM	Rotundone-Low
Average path distance (GD)	4.191	4.119	5.464
Geodesic efficiency (E)	0.291	0.296	0.245
Harmonic geodesic distance (HD)	3.438	3.384	4.079
Maximal degree	23	19	30
Nodes with max degree	OTU_397	OTU_397;OTU_287	OTU_229
Centralization of degree (CD)	0.149	0.135	0.152
Maximal betweenness	2821.18	1556.448	5154.316
Nodes with max betweenness	OTU_397	OTU_397	OTU_229
Centralization of betweenness (CB)	0.298	0.207	0.306
Maximal stress centrality	17244	10135	16962
Nodes with max stress centrality	OTU_397	OTU_287	OTU_229
Centralization of stress centrality (CS)	1.813	1.37	0.995
Maximal eigenvector centrality	0.395	0.384	0.431
Nodes with max eigenvector centrality	OTU_397	OTU_287	OTU_229
Centralization of eigenvector centrality (CE)	0.344	0.33	0.401
Density (D)	0.025	0.03	0.016
Reciprocity	1	1	1
Transitivity (Trans)	0.024	0.057	0.168
Connectedness (Con)	0.857	0.901	0.618
Efficiency	0.979	0.976	0.982
Hierarchy	0	0	0
Lubeness	1	1	1

Figure S1. Vineyard Field layout showing the sampling points; (A) non-mulched and (B) mulched area

(A) Non-mulched

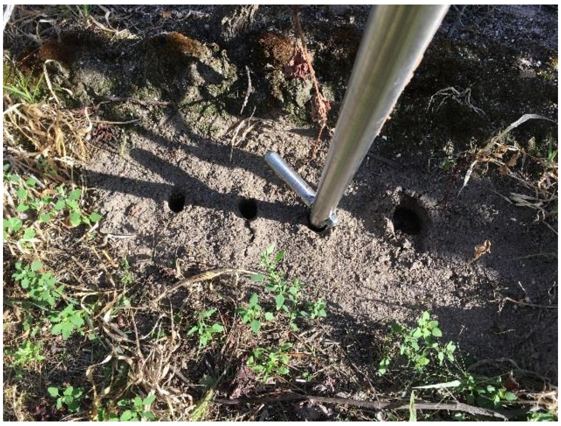

(B) Mulched

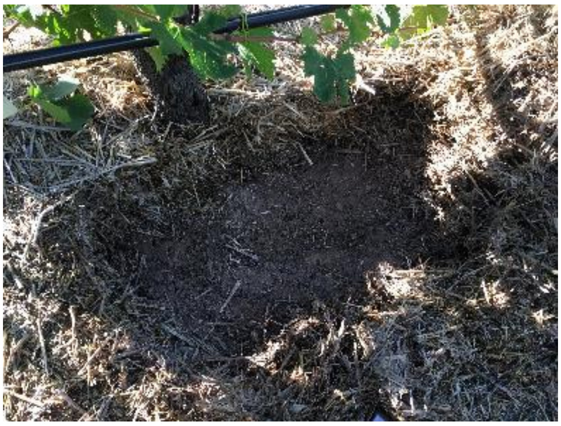

Figure S2. Effect of mulch application on the composition of bacterial and fungal communities in the Medium and High rotundone zone soils; dbRDA plots (A and B) and taxonomic composition (C and D).

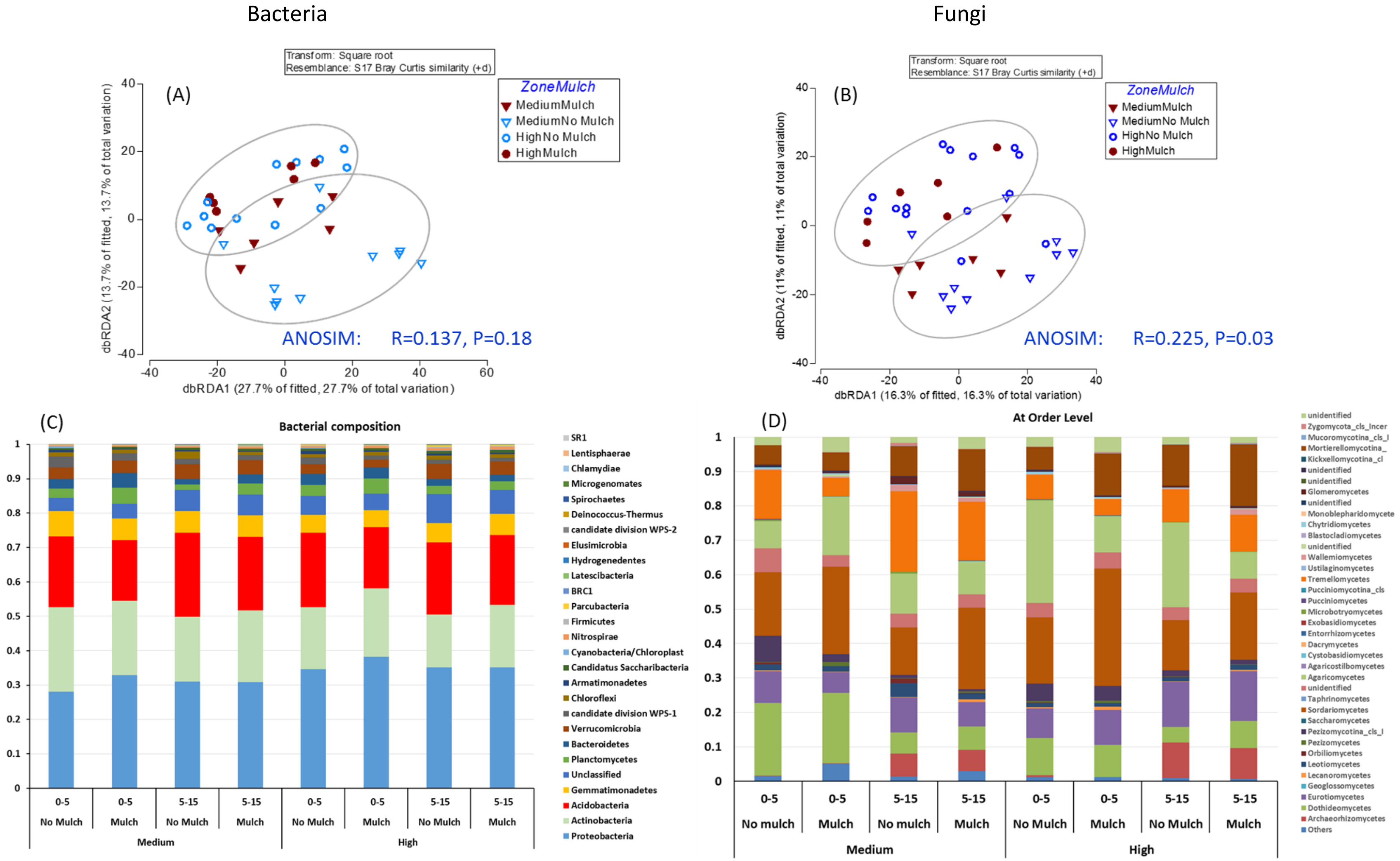

Figure S3. Effect of mulching on the microbial catabolic profiles in the surface soil samples from the Medium and High rotundone-zones; (A) Principle component analysis plot showing the dissimilarity in the catabolic diversity of soil microbial communities between with mulch and no-mulch zones and (B) heat map of the substrate use efficiency by the soil microbial communities for the various C-substrates (AWCD).

(A)

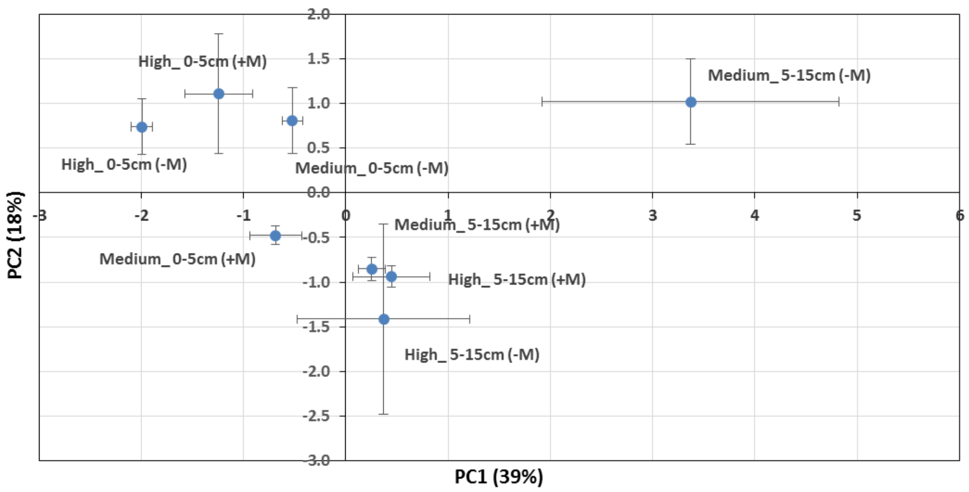

(B)

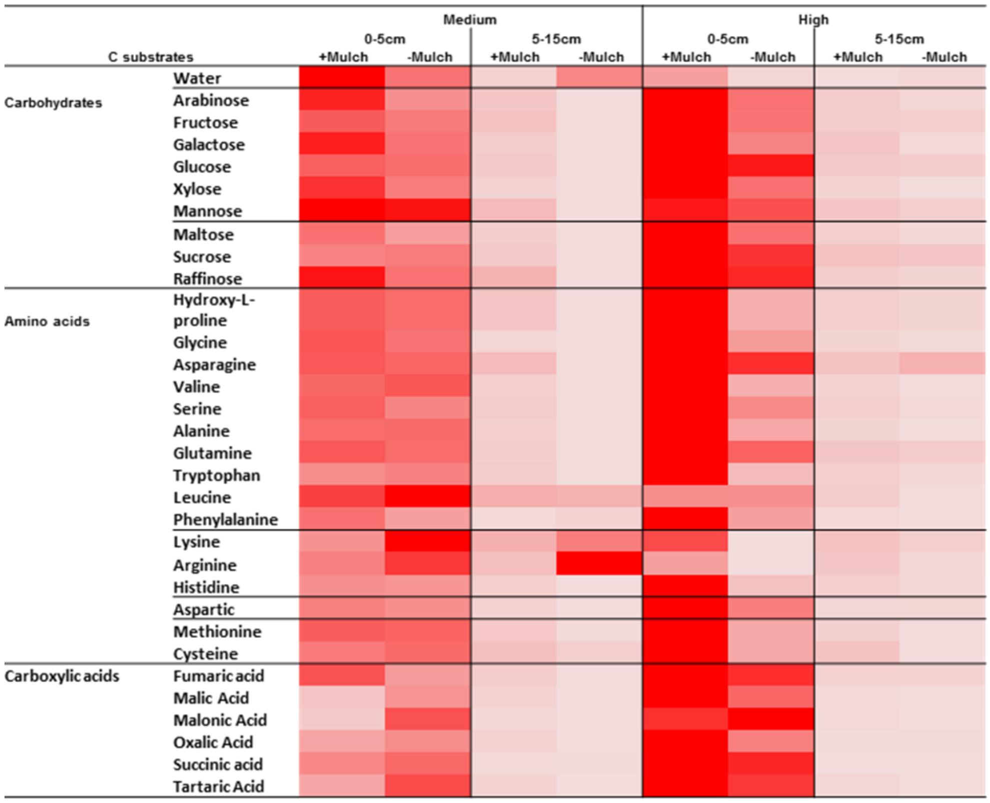

	Medium 0-5cm +Mulch	Medium 0-5cm -Mulch	Medium 5-15cm +Mulch	Medium 5-15cm -Mulch	High 0-5cm +Mulch	High 0-5cm -Mulch	High 5-15cm +Mulch	High 5-15cm -Mulch	LSD (P<0.05)
Average Metabolic Response (AMR)	0.73	0.69	0.17	0.05	1.46	0.88	0.14	0.11	not sig
AMR (Carbohydrates)	1.02	0.74	0.22	0.05	1.66	1.08	0.19	0.14	0.21
AMR (Amino acids)	0.65	0.57	0.16	0.06	1.24	0.62	0.13	0.12	not sig
AMR (Carboxylic acids)	0.49	0.92	0.12	0.03	1.71	1.26	0.08	0.06	not sig
Community Metabolic diversity (CMD)	26.44	29.67	18.11	4.00	28.11	25.89	15.56	9.89	5.2

low high

Figure S4. Comparison of Rotundone-high without mulch (A) and rotundone-high (B) networks. Circles represent nodes whose size indicates connectivity, node color represents taxonomy at the phyla level. Edges indicate co-occurrence between nodes colored either blue for positive or red for negative. Each circular grouping is a module. Numbers within modules correspond to numbers indicated in the hierarchical clustering. Hierarchical clustering based on Pearson correlations among module-eigengenes and a heatmap of module eigengenes of the rotundone-high without mulch (C) and rotundone-high (D) networks.

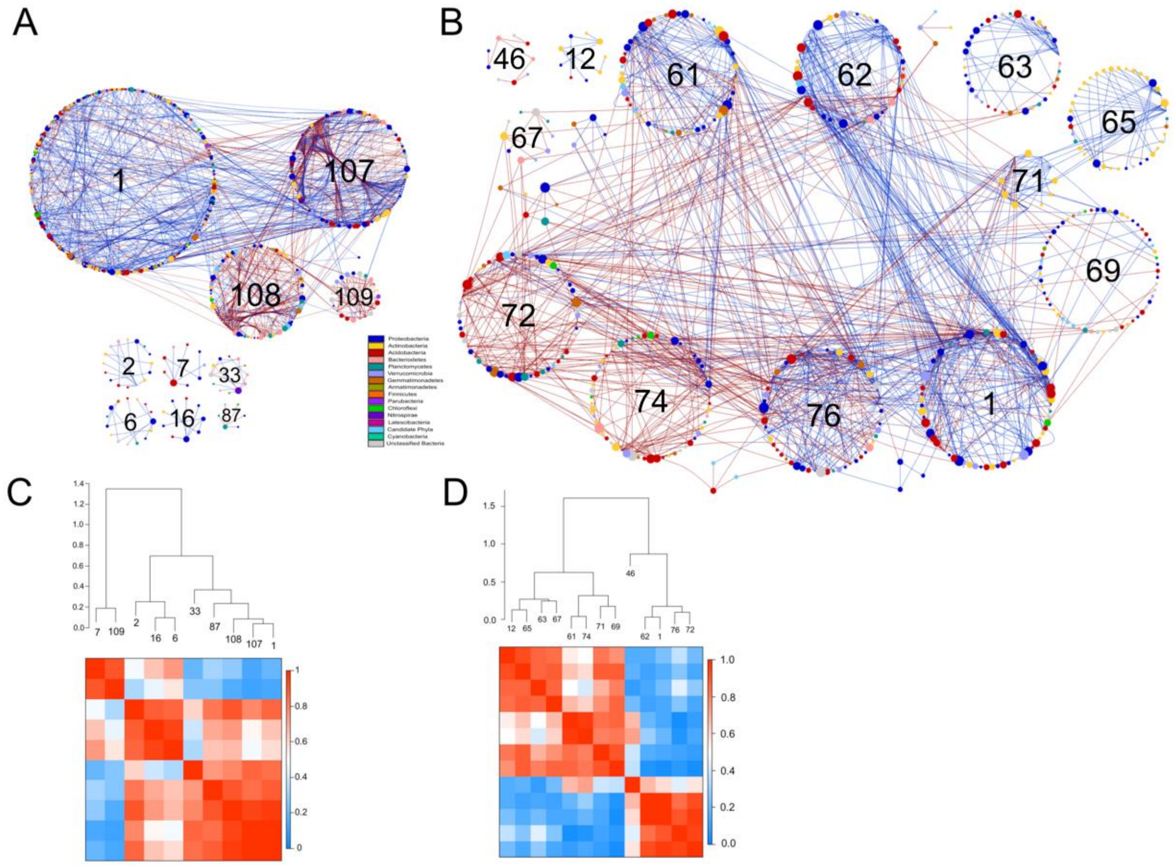

Figure S5. Comparison of rotundone-high without mulch (A) and rotundone-high (B) networks. Circles represent nodes whose size indicates connectivity, node color represents taxonomy at the phyla level. Edges indicate co-occurrence between nodes colored either blue for positive or red for negative. Each circular grouping is a module. Numbers within modules correspond to numbers indicated in the hierarchical clustering. Hierarchical clustering based on Pearson correlations among module-eigengenes and a heatmap of module eigengenes of the (C) rotundone-high without mulch and (D) rotundone-high networks.

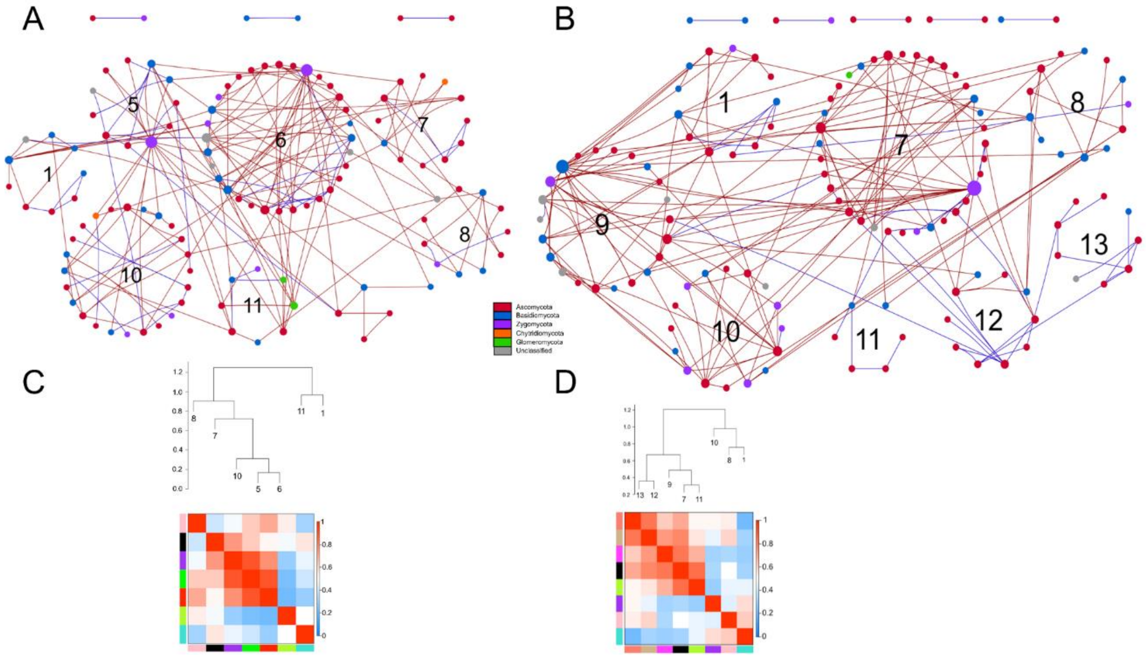

Figure S6. Correlations of module-eigengenes and environmental factors for the rotundone-high (A) and rotundone-high without mulch (B) bacterial networks. The numbers in each plot are the correlation coefficient (r) and significance (p) is in parentheses.

(A) Non-mulched

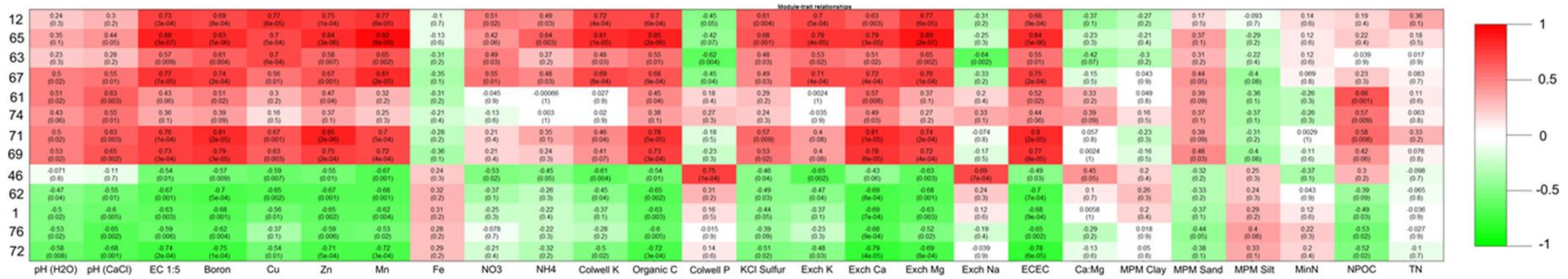

(B) Mulched

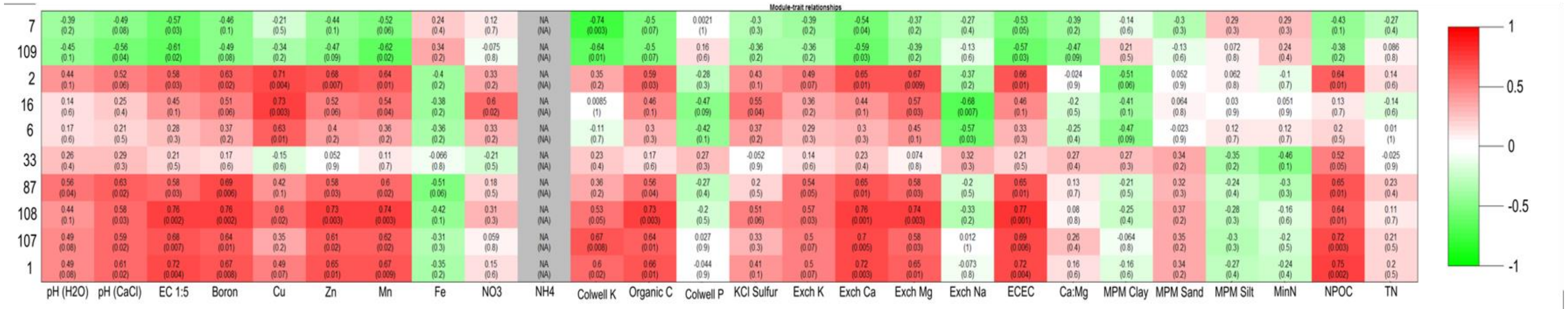

Figure S7. Correlations of module-eigengenes and environmental factors for the rotundone-high (A), rotundone-high without mulch (B) and rotundone-low fungal networks. The numbers in each plot are the correlation coefficient (r) and significance (p) is in parentheses.

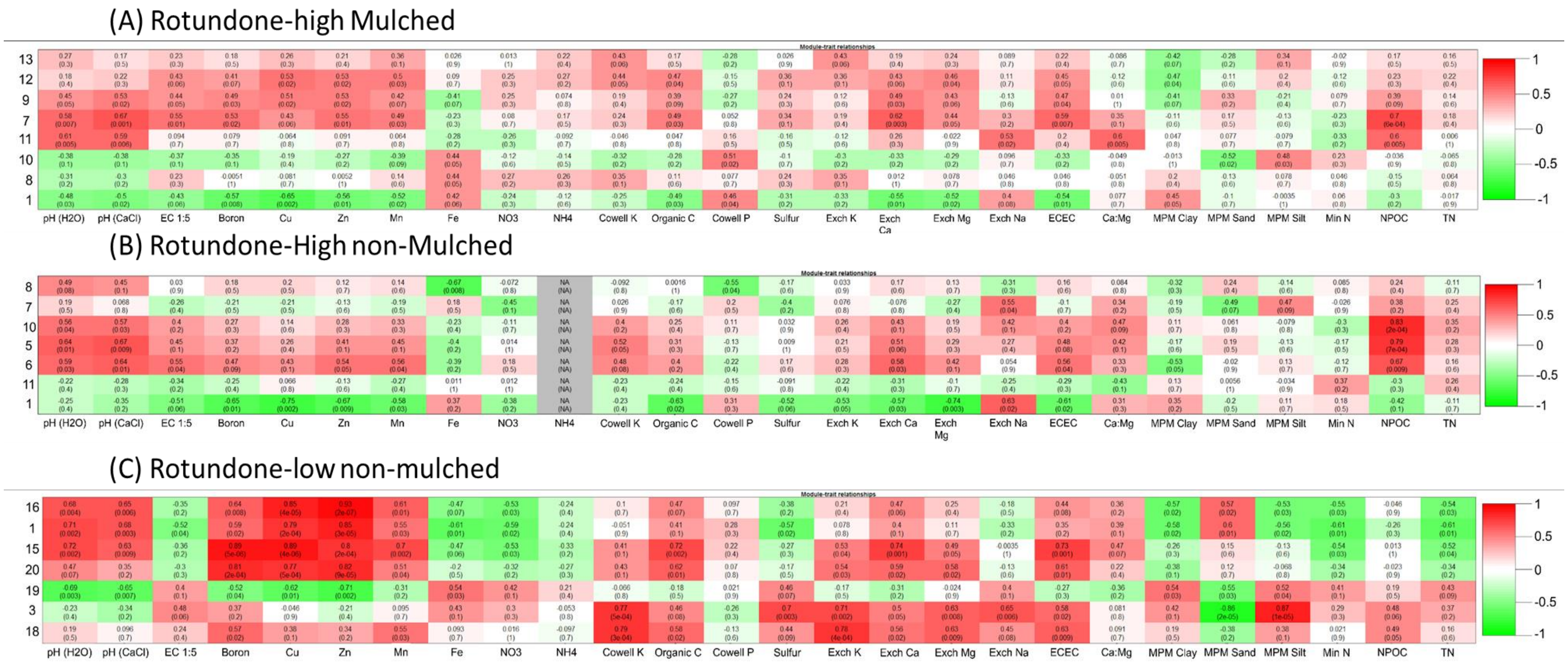

Figure S8. Topological roles of OTUs based on distribution of nodes on Zi (within module) vs. Pi (among module) connectivity scatter plots for (A) Bacteria and (B) Fungi. Each color represents an OTU from three networks.

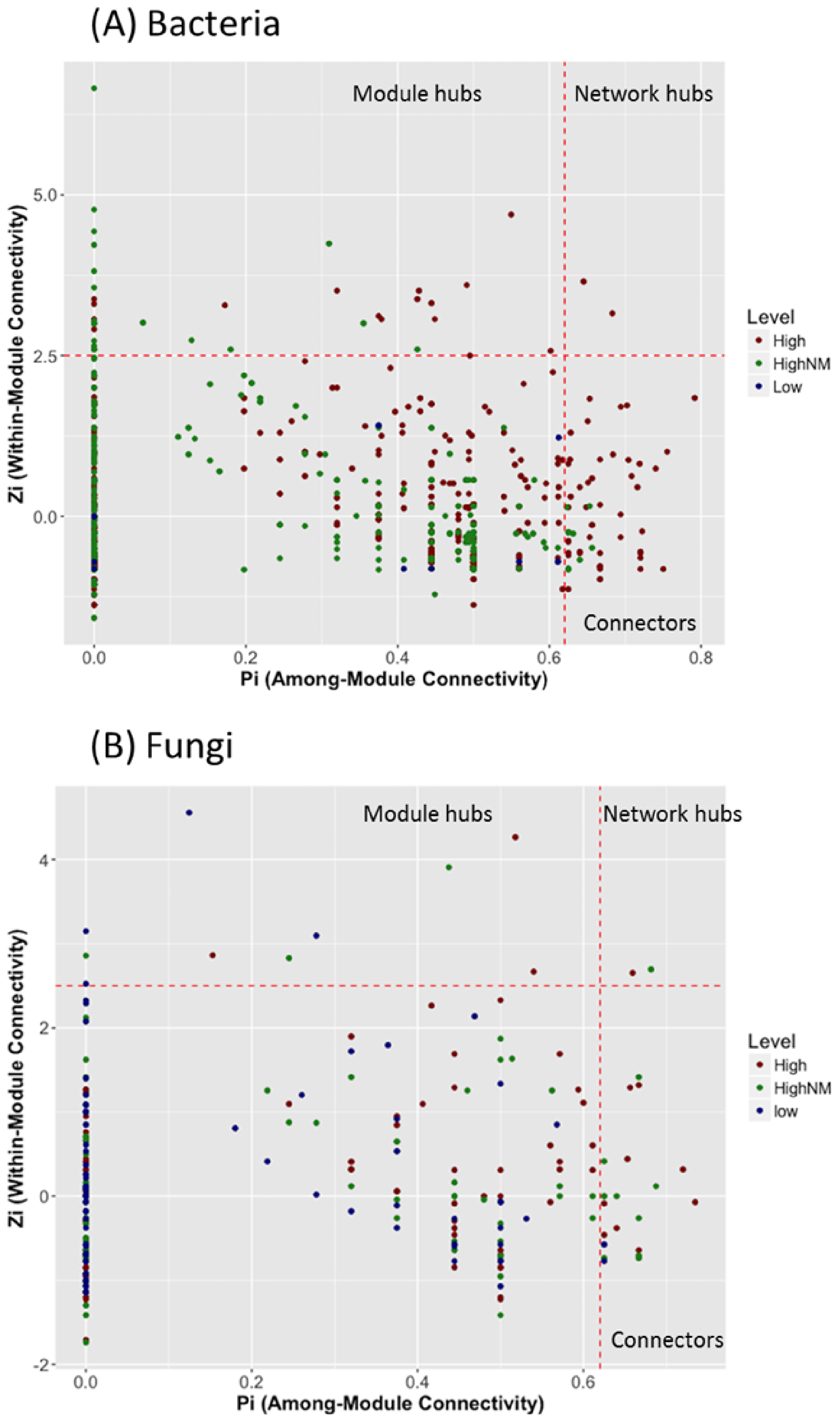

Methods:

Catabolic diversity and potential

Microbial catabolic response (CO_2 production to the addition of specific C-containing substrates) and diversity was measured through carbon substrate utilization profiles of soil microbial communities (‘community-level physiological profiles’, CLPP) using a modified Microresp® technique (Campbell et al., 2003) adjusted for Australian soils (Knox et al., 2009). Soil moisture of the sieved (2 mm) field samples was first adjusted to 50% field capacity and pre-incubated at 25°C in the dark for 48 h, before delivery into three replicate deep well MicroResp® trays per sample. The amount of CO_2 evolved after addition of 31 C-substrates was measured using absorbance (at 590 nm) of cresol red colour change from 0 to 5 hours and calculated from a standard curve. Three different substrate groups used represented C compounds that are generally found in agricultural soils and included carbohydrates (9), amino acids (16) and carboxylic acids (6) and each substrate concentration was set according to an equivalent C content between all substrate solutions (Campbell et al. 2003; Knox et al. 2009). Average well colour development (AWCD) values for each sample were calculated using the CO_2-evolved data for all substrates and water, and the respiration values for different substrates were normalized against average well colour development for each sample (Garland, 1997). The average metabolic response reflecting the overall functional capability of soil heterotrophic microbial communities was calculated from the substrate induced respiration values for each substrate (respiration with substrate added minus no-carbon value). Community metabolic diversity was estimated as the number of substrates out of 31 utilized by the microbial community (using a 0.25 threshold for optical density).
